# Deep Learning Enables Rapid Identification of a New Quasicrystal from Multiphase Powder Diffraction Patterns

**DOI:** 10.1002/advs.202304546

**Published:** 2023-11-14

**Authors:** Hirotaka Uryu, Tsunetomo Yamada, Koichi Kitahara, Alok Singh, Yutaka Iwasaki, Kaoru Kimura, Kanta Hiroki, Naoya Miyao, Asuka Ishikawa, Ryuji Tamura, Satoshi Ohhashi, Chang Liu, Ryo Yoshida

**Affiliations:** ^1^ Department of Applied Physics Tokyo University of Science 6‐3‐1 Niijuku, Katsushika‐ku Tokyo 125‐8585 Japan; ^2^ Department of Materials Science and Engineering National Defense Academy 1‐10‐20 Hashirimizu, Yokosuka Kanagawa 239‐8686 Japan; ^3^ Department of Advanced Materials Science The University of Tokyo 5‐1‐5 Kashiwanoha, Kashiwa Chiba 277‐8561 Japan; ^4^ Electron Microscopy Unit, Research Network and Facility Services Division National Institute for Materials Science 1‐2‐1 Sengen Tsukuba Ibaraki 305‐0047 Japan; ^5^ Thermal Energy Materials Group, Research Center for Materials Nanoarchitectonics National Institute for Materials Science 1‐2‐1 Sengen, Tsukuba Ibaraki 305‐0047 Japan; ^6^ Department of Materials Science and Technology Tokyo University of Science 6‐3‐1 Niijuku, Katsushika‐ku Tokyo 125‐8585 Japan; ^7^ Research Institute of Science and Technology Tokyo University of Science 6‐3‐1 Niijuku, Katsushika‐ku Tokyo 125‐8585 Japan; ^8^ Institute of Multidisciplinary Research for Advanced Materials Tohoku University 2‐1‐1 Katahira, Aoba‐ku, Sendai Miyagi 980‐8577 Japan; ^9^ The Institute of Statistical Mathematics Research Organization of Information and Systems 10‐3 Midori‐cho, Tachikawa Tokyo 190‐8562 Japan; ^10^ Department of Statistical Science The Graduate University for Advanced Studies 10‐3 Midori‐cho, Tachikawa Tokyo 190‐8562 Japan

**Keywords:** deep neural networks, icosahedral quasicrystals, machine learning, phase‐identification, powder X‐ray diffraction

## Abstract

Since the discovery of the quasicrystal, approximately 100 stable quasicrystals are identified. To date, the existence of quasicrystals is verified using transmission electron microscopy; however, this technique requires significantly more elaboration than rapid and automatic powder X‐ray diffraction. Therefore, to facilitate the search for novel quasicrystals, developing a rapid technique for phase‐identification from powder diffraction patterns is desirable. This paper reports the identification of a new Al–Si–Ru quasicrystal using deep learning technologies from multiphase powder patterns, from which it is difficult to discriminate the presence of quasicrystalline phases even for well‐trained human experts. Deep neural networks trained with artificially generated multiphase powder patterns determine the presence of quasicrystals with an accuracy >92% from actual powder patterns. Specifically, 440 powder patterns are screened using the trained classifier, from which the Al–Si–Ru quasicrystal is identified. This study demonstrates an excellent potential of deep learning to identify an unknown phase of a targeted structure from powder patterns even when existing in a multiphase sample.

## Graphical abstract

An automatic and accurate phase‐identification method for an icosahedral quasicrystal from powder X‐ray diffraction patterns is developed. As a result, a new Al–Si–Ru icosahedral quasicrystal is successfully identified from powder diffraction patterns even in a multiphase sample.

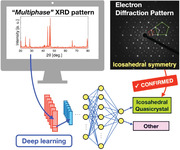

## 1 Introduction

Powder X‐ray diffraction (PXRD) is a widely used mature technology for the identification of crystalline materials. However, the analysis of PXRD patterns is challenging when a sample exists in the form of a multiphase mixture. In such cases, phase‐identification involves several rule‐based protocols that rely upon the experience and intuition of highly trained experts. Recent advances in deep learning technologies have led to remarkable performances in the analysis of PXRD patterns, including phase‐identification^[^ ^1^, ^2^, ^3^, ^4^, ^5^ ^]^ and symmetry‐classification.^[^ ^6^, ^7^, ^8^ ^]^ For example, Lee et al. applied a convolutional neural network (CNN) to determine the volume fractions of already‐known phases that exist in multiphase inorganic compounds from the PXRD patterns.^[^ ^2^ ^]^ The key to this workflow was the generation of realistic artificial multiphase diffraction patterns for the training dataset. Schuetzke et al. provided a well‐established framework for generating artificial diffraction patterns, demonstrating that the variation in unit‐cell parameters is crucial to increase the discriminative power in phase‐identification.^[^ ^9^ ^]^ However, to the best of our knowledge, the identification of an unknown phase from multiphase samples has not been successfully performed before. In this study, we aim to establish a machine learning (ML) workflow for phase‐identification of an *unknown* phase of a targeted structure type, that is icosahedral quasicrystal (i‐QC), using *multiphase* diffraction patterns.

Quasicrystals (QCs) are long‐range ordered solids that exhibit self‐similarity in their single‐grain diffraction patterns; however, their scaling ratio is incompatible with translational symmetry.^[^ ^10^ ^]^ Since the discovery of an Al–Mn i‐QC,^[^ ^11^ ^]^ QCs have been identified in a wide variety of materials, including alloys,^[^ ^11^, ^12^, ^13^ ^]^ liquid crystals,^[^ ^14^ ^]^ nanoparticle assemblies,^[^ ^15^ ^]^ mesoporous silica,^[^ ^16^ ^]^ colloidal crystals,^[^ ^17^ ^]^ polymers,^[^ ^18^ ^]^ metal oxides,^[^ ^19^, ^20^ ^]^ minerals,^[^ ^21^, ^22^ ^]^ and a metal organic framework.^[^ ^23^ ^]^ The observation of novel physical properties, such as quantum criticality in Au–Al–Yb i‐QC,^[^ ^24^ ^]^ superconductivity in Al–Mg–Zn i‐QC,^[^ ^25^ ^]^ and very recent long‐range magnetic order (ferro‐ and ferrimagnetism) in Au–Ga–(Gd,Tb)^[^ ^26^ ^]^ and Au–Ga–Dy^[^ ^27^ ^]^ i‐QCs, has always been accomplished by the discovery of new QCs. However, the search for unknown QCs has relied on time‐consuming, manual trial‐and‐error work based on human intuition; therefore, accelerating the discovery of new QCs is of enormous importance in advancing the study of quasiperiodic materials. In this context, Liu et al. recently developed an ML algorithm that can predict QC‐forming chemical compositions with ultra‐high accuracy.^[^ ^28^, ^29^ ^]^ Since it is desirable to conduct numerous synthetic experiments based on high‐throughput screening across extensive libraries of candidate materials using such an ML model, the development of rapid QC phase‐identification technologies is necessary to characterize a large number of samples that are mass‐produced in laboratory experiments. Currently, the only existing method that can be applied to the phase‐identification of i‐QCs from multiphase PXRD patterns is the scheme proposed by Lu et al.^[^ ^30^ ^]^ However, their method is applicable only in cases where an i‐QC phase is dominant in the sample.

We devised a versatile deep learning methodology that can detect the presence of an i‐QC phase, both known and unknown, from intricate multiphase PXRD pattern, even when the i‐QC phase is not dominant in the multiphase mixture. A binary classifier was constructed using CNNs that determine whether an i‐QC phase is present/absent in a sample with its PXRD pattern. The classifier was trained on the synthetic multiphase diffraction patterns, and its identification performance was evaluated based on both synthetic patterns and our in‐house database of actual patterns. The trained classifier was then applied to screen 440 measured diffraction patterns of unknown materials in five alloy systems. This screening indicated the presence of an unknown i‐QC phase in multiphase Al–Si–Ru alloys, which was subsequently confirmed in transmission electron microscopy (TEM) observations.

## 2 Results and Discussion

### 2.1 Building and Training the Binary Classifier

**Figure 1:**
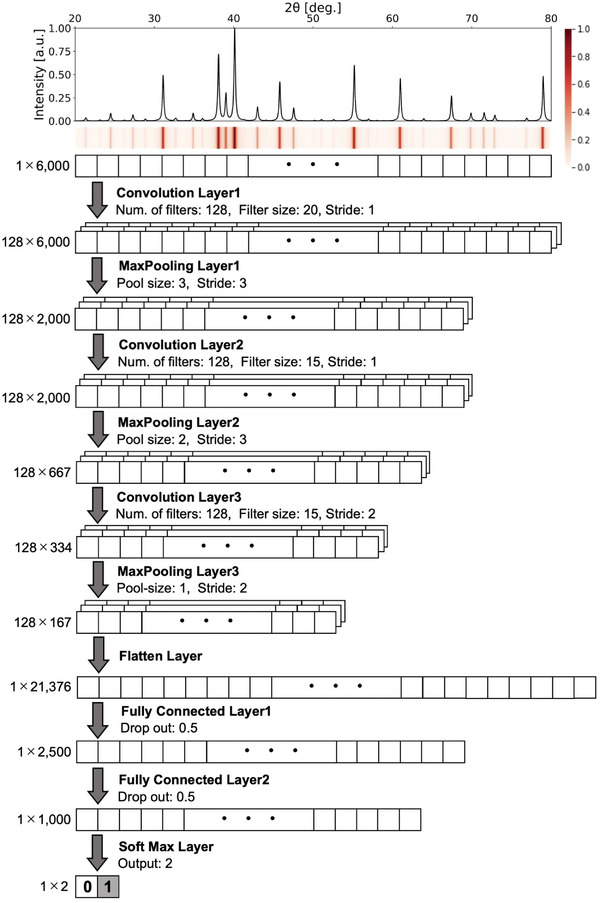
A CNN architecture for phase‐identification from the PXRD patterns. The model is composed of an input layer, three pairs of convolution and max‐pooling layers, a flatten layer, two fully connected layers, a softmax layer, and an output layer. The number of neurons in each layer are given along with the hyperparameters, the number of filters, the filter size, the stride, the pool‐size, the dropout rate, and the number of neurons in each layer.

**Figure 2:**
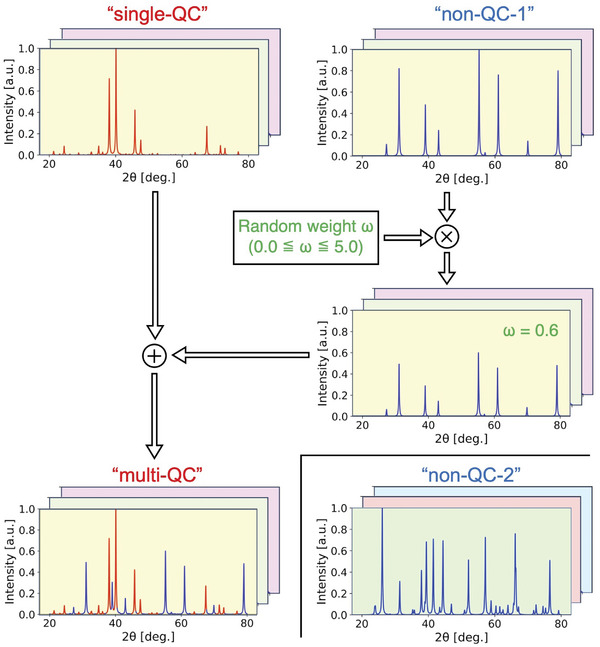
Flowchart outlining preparation of the artificial PXRD patterns for model training. Based on the structural model of the i‐QC in Figure 3d–f, single‐phase diffraction patterns of the i‐QC (”single‐QC”) with variation in the icosahedral lattice constant, the constituent elements, and the peak widths were generated. Each ”single‐QC” pattern was then added to one of the patterns generated randomly (”non‐QC‐1”) with a mixing weight ω selected randomly in the range of 0.0–5.0 (”multi‐QC”). The ”non‐QC‐2” set was generated in the same manner as ”others‐1”, with the exception that the number of “strong” peaks was selected randomly from a range of 5–35. All patterns were generated in a 2θ‐range between 20 and 80° with a Δ(2θ) interval equal to 0.01°. The peak positions and heights in each pattern were convoluted using the Lorentzian profile function.

**Figure 3:**
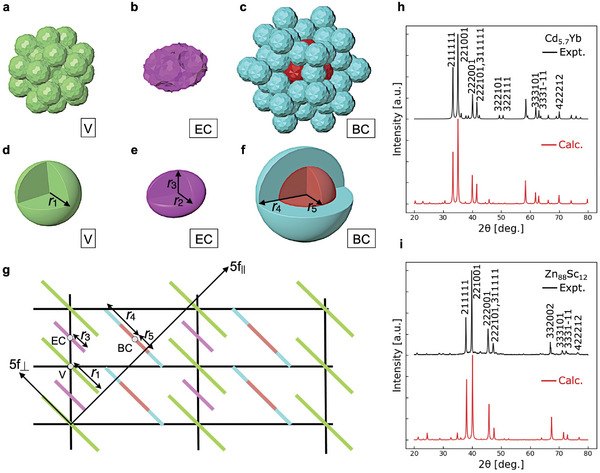
The simple 6D structure model of an i‐QC based on the Cd_5.7_Yb model.^[^ ^34^ ^]^ The three independent occupation domains (ODs) in the Cd_5.7_Yb model are located at a) the vertex, b)the edge‐center, and c) the body‐center positions of the 6D lattice. These ODs were simplified as follows: d) a sphere with a radius *r*_1_ at the vertex, e) a spheroid with an equatorial radius of *r*_2_ and a distance of *r*_3_ from the center to the pole at the edge‐center, and f) a sphere with a radius *r*_4_. The sphere in (f) is decomposed into two parts, that is, a central spherical shell with a radius of *r*_5_ and the remainder. g) The fivefold section of the 6D structure model. The bars indicate the five‐fold section of the ODs, while 5f_‖_ and 5f_⊥_ indicate the fivefold axes in **E**_‖_ and **E**_⊥_, respectively. The PXRD patterns for the h) Cd_5.7_Yb and i) Zn_88_Sc_12_ i‐QCs are compared to those computed based on the ODs in (d–f). The six indices are given using an indexing scheme for i‐QCs.^[^ ^35^ ^]^

The icosahedral lattice constant *a* of the i‐QCs found so far ranges from approximately 0.45 (Al_65_Cu_20_Fe_15_ i‐QC^[^ ^31^ ^]^) to 0.58 nm (Cd‐Mg‐Yb i‐QC^[^ ^32^ ^]^). Thus, the classifier was composed of 80 different CNNs, where the *i*‐th CNN determined the presence/absence of i‐QCs with an icosahedral lattice constant given by *a*_*i*_ = 0.4000 + 0.0025*i* nm (*i* = 0, 1, …, 79). This facilitates the investigation of any arbitrary alloy for the presence of an i‐QC phase. The model architecture and hyperparameters, which were common to these CNNs, were designed to optimise the classification accuracy in hold‐out validation through Bayesian optimisation^[^ ^33^ ^]^ (see **Figure** **1**). To train each CNN, a dataset of artificial multiphase PXRD patterns was prepared. Each pattern was synthesized by mixing two patterns denoted as “single‐QC” for single‐phase i‐QCs and “non‐QC” for other phases (see **Figure** **2**). In accordance with a previous study showing that the variation of unit‐cell parameters in a training set has a significant impact on the performance of the phase‐identification task,^[^ ^9^ ^]^ the “single‐QC” patterns were generated using an icosahedral lattice constant varied randomly between *a*_*i*_ and *a*_*i*_ + 0.0025 nm. A total of 30,000 “single‐QC” patterns were generated using a simple i‐QC model as shown in **Figure** **3**. Two different sets of “non‐QC” were also generated using a rule‐based procedure, each containing 30 000 patterns (“non‐QC‐1” and “non‐QC‐2”). To create the multiphase i‐QC diffraction patterns using these sets (hereafter referred to as “multi‐QC”), each “single‐QC” was mixed with a randomly selected pattern of “non‐QC‐1” with a mixing weight sampled between 0.0 and 5.0. The “non‐QC‐2” set was used as the negative instance set, denoted by “non‐QC”, with respect to the positive instances in “multi‐QC”.

Each CNN was trained using the “multi‐QC” and “non‐QC‐2” datasets. Its prediction performance was then evaluated using an unseen dataset of 6,000 additionally generated artificial patterns. The prediction accuracy reached 98.9% (Table S1, Supporting Information). Furthermore, the prediction performance was evaluated using an unseen dataset of 424 manually annotated experimental patterns. The accuracy reached 92.2% (Table S1, Supporting Information). Here, a classification probability, denoted by *p*, of the classifier was defined to be the highest value among all probabilities given by the CNNs. The performance was also examined based on two additional metrics, namely recall and precision. Recall is a measure of the number of i‐QC instances accurately predicted by the classifier, while precision is the measure of the number of instances predicted to be i‐QC that are actually true. The recall and precision reached 0.989 and 0.990 for the artificial dataset and 0.959 and 0.700 for the experimental dataset, respectively. Although the precision, with respect to the experimental dataset, was slightly lower than that of the artificial dataset, the recall was maintained at a significantly high level. Importantly, during high‐throughput screening, it is necessary to avoid overlooking the presence of QCs; therefore, it is crucial to achieve a high level of recall even if the precision is slightly lower. These results indicate that our classifier proved to be useful in practice. Moreover, experimental PXRD patterns of known i‐QCs were successfully identified by the trained classifier (Table S2, Supporting Information). It is interesting to note that the classifier could identify the i‐QCs of three different families, i.e., Bergman‐, Mackay‐ ,and Tsai‐types, although a simple structure model was used in the generation of the “single‐QC” patterns. Broadly speaking, the powder diffraction patterns of these i‐QCs is similar to each other; therefore, the classification performance of the present model is not sufficient to distinguish different types of the i‐QCs. The classification of different types of i‐QCs is one of our research topics in the feature.

For comparison, we constructed deep neural network models with a single CNN as shown in Figure 1 to determine the presence/absence of i‐QCs with an icosahedral lattice constant ranging from 0.4 to 0.6 nm. The prediction performance of these models was found to be much lower than that of the above binary classifier using multi‐CNN, shown by the following.

First, the single CNN was trained with a dataset of 30 000 patterns of “single‐QC” and 30 000 patterns of “non‐QC”, and the prediction performance of the model evaluated using an unseen dataset of 10 000 additionally generated artificial patterns, that is 5,000 “single‐QC” and 5,000 “non‐QC” patterns. The prediction performance was most likely perfect, with accuracy, recall, and precision equal to 99.67, 99.96, and 99.38%, respectively (see Table S3a, Supporting Information). The performance decreased however when evaluated using an unseen dataset of artificial patterns of multiphase mixture, that is 5,000 “multi‐QC” and 5,000 “non‐QC” patterns (see Table S3b, Supporting Information), where the accuracy and recall decreased to 57.92% and 16.36%, respectively. Although the precision remained high (96.92%), the recall was too low for practical use.

Second, the model was trained with a dataset of artificial multiphase patterns, i.e., 30 000 “multi‐QC” and 30 000 “non‐QC” patterns. The accuracy, recall and precision respectively reached 79.30, 75.54, and 81.68% against an unseen dataset of 10 000 additionally generated multiphase patterns (see Table S3c, Supporting Information). Although the prediction performance improved over the above single CNN model, it was beyond the binary classifier using multi‐CNN.

### 2.2 Screening 440 Experimental Powder XRD Patterns and Characterization of Candidate Materials

Subsequently, the trained classifier, which is composed of multi‐CNNs, was employed to screen 440 entries of unlabeled PXRD patterns. These laboratory data were accumulated for the purpose of material exploration in six alloy systems, including Al–Si–Ru,^[^ ^36^ ^]^ Al–Fe–Ir, Al–Mn–Ir, Al–Cu–Pt, Al–Pt–Co, and Al–Cu–Ir; therefore, most samples were likely to be multiphase mixtures. The presence or absence of i‐QC was determined by the magnitude of *p* being greater or <0.950. Furthermore, each pattern was classified into one of the following four classes according to the *p*: A (*p* > 0.999), B (0.990 < *p* ⩽ 0.999), C (0.950 < *p* ⩽ 0.990), and D (*p* ⩽ 0.950).

**Figure 4:**
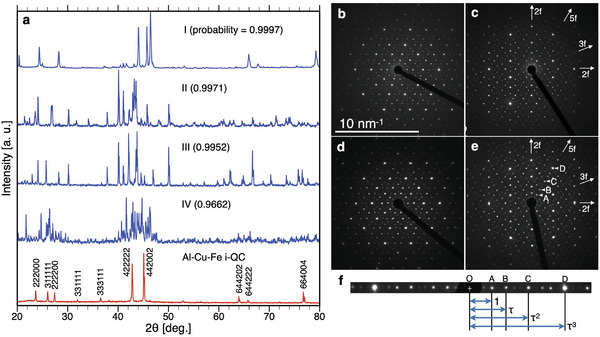
a) Measured PXRD patterns of four different Al–Si–Ru alloys. The red‐coloured pattern on the bottom is a measured diffraction pattern for a single‐phase of the Al–Cu–Fe i‐QC. The classification probabilities of including an i‐QC phase are indicated in the figure. The SAED patterns of the Al–Si–Ru samples related to powder b,c) patterns‐I and d,e) IV are shown. These patterns were taken along (b,d) the fivefold and (c,e) the twofold axes, depicting the formation of the i‐QC. e) The peaks A, B, C, and D on the twofold pattern are indexed as 200000, 111111, 311111, and 422222, respectively, according to a previous report.^[^ ^35^ ^]^ f) τ‐scaling property of the diffraction pattern.

The number of master alloys and diffraction patterns are given in **Table** **1**, together with the number of patterns classified as the prediction class ϕ={A,B,C,D}. The last column in the table is total score estimated from the diffraction pattern of each alloy system. The score is defined as score = 1/N∑_ϕ_S_ϕ_n_ϕ_, where N, n_ϕ_, S_ϕ_ are the number of diffraction patterns for each alloy system, number of patterns classified as ϕ, and partial score given to each pattern classified as ϕ, respectively. We set S_A_, S_B_, S_C_, and S_D_ equal to 3, 2, 1, and 0, respectively. Among the six alloy systems, the highest score was obtained in the Al–Si–Ru alloy system, where the screening result yielded 4, 16, and 53 patterns classified as A, B, and C, respectively. The alloy composition of the Al–Si–Ru samples is shown in a ternary diagram in Figure S1 (Supporting Information) with the predicted class of its corresponding diffraction pattern. It was found that the compositions of the samples exhibiting a diffraction pattern classified as B or C were distributed around those classified as A. Four of these candidate patterns were then selected (denoted by I to IV in **Figure** **4**a); pattern‐I was classified as class A, patterns‐II and III as B, and pattern‐IV as C. The presence of i‐QCs in these alloys (hereafter denoted as ASR(I) to ASR(IV)) was then examined in detail through scanning electron microscopy (SEM).

**Table 1:** Details of the 440 PXRD patterns of unknown materials. Number of master alloys is shown in the 2nd column for each alloy system shown in the 1st column. The number of PXRD patterns is given in the 3rd column and number of patterns classified to as A, B, C, or D is given in the 4–7th columns. The last column is the total score estimated for the PXRD patterns of each alloy system.

Alloy system	N. of master alloy	N. of data	A	B	C	D	Score
Al–Si–Ru	79	237	4	16	53	164	0.401
Al–Fe–Ir	52	130	2	13	15	100	0.362
Al–Mn–Ir	12	42	0	0	2	40	0.048
Al–Cu–Pt	8	21	0	0	0	21	0.000
Al–Pt–Co	8	8	0	0	1	7	0.125
Al–Cu–Ir	2	2	0	0	0	2	0.000
total	161	440	6	29	71	334	

For pattern‐I, the majority of peaks were assigned to a primitive i‐QC with *a* equal to 0.4353 nm. However, some intense peaks were not assigned to an i‐QC. The SEM observations revealed that three different phases were present in ASR(I), and their compositions were determined to be Al_43_Si_32_Ru_25_, Al_26_Si_34_Ru_20_, and Al_38_Si_42_Ru_20_ using energy dispersive X‐ray (EDX) spectroscopy. Moreover, through electron back‐scattered diffraction (EBSD), we confirmed that the first phase exhibited Kikuchi patterns with icosahedral symmetry, while the others did not, indicating the presence of the i‐QC phase in the multiphase mixture (see Figure S2, Supporting Information).

As shown in Figure 4a, the diffraction patterns‐II, III, and IV exhibit a large number of peaks that are absent in pattern‐I. Using SEM‐EDX, it was observed that ASR(II) is a single‐phase with a composition of Al_73_Si_1_Ru_26_. In addition, ASR(III) was found to be composed of two different phases with compositions of Al_69_Si_5_Ru_26_ and Al_61_Si_3_Ru_36_. From their Kikuchi patterns, it was confirmed that these phases are not i‐QC. For pattern‐IV, ASR(IV) was found to be composed of three different phases with compositions of Al_44_Si_31_Ru_25_, Al_55_Si_17_Ru_28,_ and Al_53_Si_23_Ru_24_. It was confirmed that the first phase exhibited icosahedral Kikuchi patterns, whereas the others did not, indicating that the i‐QC phase was present in the multiphase mixture (see Figure S3, Supporting Information).

To further examine the presence of i‐QC phase, TEM observations were performed for ASR(I) and ASR(IV). Figure 4b–e shows the selected‐area electron diffraction (SAED) patterns of these alloys with incidence along the fivefold and twofold rotational symmetry axes, indicating the presence of the i‐QC phase in these samples. To the best of our knowledge, there are no previous reports on the presence of i‐QC in Al–Si–Ru alloy systems. Importantly, these SAED patterns allowed characterization of the i‐QC phase in more detail. More specifically, the twofold diffraction pattern was found to exhibit τ‐scaling (see Figure 4f), where τ is the golden mean equal to $\left(1+\sqrt{5}\right)/2$; this observation indicates that these i‐QC phases form a face‐centered type superstructure with a doubled lattice parameter, *a*′ = 2*a* = 0.8706 nm.

The alloy composition of Al–Si–Ru i‐QC reported herein differs from those of known i‐QCs. To date, stable face‐centered i‐QCs have been observed in Al transition metal systems, such as Al_65_Cu_20_Fe_15_ (*a*′ = 0.890 nm),^[^ ^31^ ^]^ Al_65_Cu_20_Ru_15_ (0.906 nm), Al_65_Cu_20_Os_15_ (0.902 nm),^[^ ^37^ ^]^ Al_70_Pd_20_Mn_10_ (0.914 nm),^[^ ^38^, ^39^ ^]^ Al_70_Pd_20_Tc_10_ (0.921 nm),^[^ ^40^ ^]^ and Al_70_Pd_20_Re_10_ (0.922 nm).^[^ ^38^, ^41^ ^]^ Therefore, an Al concentration of approximately 43 at.% in the Al–Si–Ru i‐QC is significantly lower than the corresponding values for the previously synthesized i‐QCs. Furthermore, the Al–Si–Ru i‐QC contained approximately 30 at.% of Si; no Al‐based i‐QC containing such a large amount of Si has been previously reported, with the exception of the Si_61_Cu_30_Ca_7_Fe_2_ i‐QC, which was observed in a sample created in the first atomic bomb test.^[^ ^42^ ^]^ Moreover, the lattice constant *a*′ of the Al–Si–Ru i‐QC is the smallest among those of the above‐mentioned Al‐based i‐QCs, with a difference of at least 0.02 nm.

Although pattern‐I is similar to the PXRD pattern of the Al–Cu–Fe i‐QC (Figure 4a), several differences were observed. First, the second intense peak at 2θ = 45.72° in pattern‐I was absent in the Al–Cu–Fe i‐QC, and this peak originated from the secondary phases in ASR(I). Second, the peak positions were shifted in pattern‐I owing to the large difference in the lattice constants (approximately 0.2 nm). Third, the 311111 peak observed at 2θ = 26.06° in the diffraction pattern of the Al–Cu–Fe i‐QC was not observed in pattern‐I. Since this peak corresponds to a strong superstructure reflection that has been observed in diffraction patterns for known Al‐based face‐centered i‐QCs, the presence of this peak forms the basis for judging whether the i‐QC phase is present/absent in the search for new Al‐based i‐QCs containing transition metals. In rule‐based identification, the aforedescribed differences would lead to human experts misjudging the absence of the i‐QC phase in ASR(I). As regards pattern‐IV, the visual recognition of the presence of the Al–Si–Ru i‐QC is considerably difficult, even for highly skilled researchers.

## 3 Conclusion

The classifier, which is composed of multi‐CNNs and trained from artificial multiphase PXRD patterns, achieved a high discriminative power in the phase‐identification of i‐QCs. Therefore, it significantly accelerates the phase‐identification task for multiphase samples, which is one of the bottlenecks in the QC exploration process. The applicability of the current method is not limited to i‐QC analysis, but the same ML approach could be used in phase‐identification of new decagonal and dodecagonal QCs, using their prototype models.^[^ ^43^, ^44^ ^]^ Further, the present ML approach is expected to be applicable to various types of crystalline materials and facilitates the materials discovery process.

## 4 Experimental Section

### Convolutional Neural Network

The CNNs for the different lattice constants were designed with a common model architecture, as shown in Figure 1.^[^ ^45^ ^]^ Keras^[^ ^45^ ^]^ was used for implementation. This model consisted of the nine layers that define the mapping from any given diffraction pattern to the output probability of the binary classification. Each of the layers consisted of convolutional and max‐pooling layers, followed by a flatten layer, two fully connected layers, and a softmax layer.

### Hyperparameter Search

The hyperparameters of the CNN were tuned by running Optuna^[^ ^46^ ^]^ for 50 trials. The objective function to be optimized was the accuracy of the validation data, where 20% of the 60 000 training samples were randomly chosen. The search space is summarized in Table S4 (Supporting Information), and included the number of convolutional layers, number of kernels and the kernel size for each convolutional layer, number of layers and neurones for the fully connected layers, pooling size, dropout rate, and choice of optimiser. The black‐box function was optimized by performing Bayesian optimization using a tree‐structured Parzen estimator.^[^ ^33^ ^]^ The resulting values are given in Figure 1.

### Performance Measures

The classification performance was examined based on the recall and the precision with respect to the discrimination capability of “i‐QC”: recall = TP/(TP + FN) and precision = TP/(TP + FP), where TP, TN, FP, and FN denoted the occurrence numbers of true positive, true negative, false positive, and false negative instances in the prediction, respectively. The overall accuracy was defined as follows: accuracy = (TP + TN)/(TP + FN + FP + TN).

### Calculation of Powder X‐ray Diffraction Patterns for Training

Single‐phase diffraction patterns of the primitive‐type i‐QCs were generated (“single‐QC” patterns in Figure 2) based on hyperspace crystallography.^[^ ^47^, ^48^, ^49^ ^]^ In the structure generator, the atomic structure of an i‐QC was described as a 3D section of a 6D periodic structure consisting of occupation domains (ODs). Each OD represented a hypothetical 3D object in a 3D complementary space called the perpendicular space (**E**_⊥_), which was perpendicular to the 3D real space known as the parallel space (**E**_‖_).

A simple 6D structure model was constructed based on the model of Tsai‐type Cd_5.7_Yb i‐QC^[^ ^34^ ^]^ (Figure 3a–c). The Cd_5.7_Yb model consisted of ODs located at three independent positions of a 6D hyper‐cubic lattice with a 6D unit‐cell parameter (*a*_6D_) as follows: Vertex position (V) at (0,0,0,0,0,0) Edge‐center position (EC) at (1,0,0,0,0,0)/2, and Body‐center position (BC) at (1,1,1,1,1,1)/2. In this model, Yb atoms occupied the central part of the OD at the BC, and Cd atoms occupied the remainder of the OD. The resulting atomic structure possessed an icosahedral lattice constant $a\;(=a_{6D}/\sqrt{2}$) of 0.5689 nm. To reduce the computational cost of obtaining the diffraction patterns, the ODs were approximated as spheres, spheroids, or spherical shells as follows: i) The OD at V was approximated as a spherical OD with a radius of *r*_1_, ii) OD at EC was approximated as a spheroid‐shaped OD with an equatorial radius of *r*_2_ and a distance of *r*_3_ from the center to the pole, and iii) OD at BC was approximated as a spherical OD with a radius of *r*_4_ that was further decomposed into two objects, namely a central sphere with a radius of *r*_5_ and a spherical shell (see Figure 3d–g). Using this spherical 6D model, the PXRD patterns were calculated according to a previous literature approach,^[^ ^50^ ^]^ in which the parameters *r*_*i*_(*i* = 1, 2, …, 5) were optimized based on the experimental diffraction pattern of Cd_5.7_Yb i‐QC.^[^ ^51^, ^52^ ^]^ Here, it was assumed that the spherical OD at BC was occupied by Yb and the remainder of the OD was occupied by Cd. The resulting PXRD pattern well reproduced the experimental observations (Figure 3h). Moreover, when replacing the Cd and Yb atoms in the model with Zn and Sc, respectively, it was confirmed that the simulated PXRD pattern successfully reproduced the experimental diffraction pattern of Zn_88_Sc_12_ i‐QC^[^ ^53^ ^]^ (see Figure 3i).

In the generation of “single‐QC” patterns, the constituent elements of each i‐QC were randomly selected from the 60 different metal elements to generate binary, ternary, and quaternary i‐QC structures. Each pattern was calculated based on the use of Cu‐Kα_1_ radiation with Bragg–Brentano geometry in a 2θ‐range between 20

and 80°, which is a typical setup for measuring i‐QC alloys.

The “non‐QC‐1” pattern set in Figure 2 was generated based on the rule that each pattern consisted of “strong” and “weak” peaks, whose intensities were selected randomly in the ranges of 0.1–1.0 and 0.0–0.1, respectively. In addition, the number of peaks was selected randomly in the ranges of 0–30 and 0–100 for the “strong” and “weak” peaks, respectively. The “non‐QC‐2” patterns in Figure 2 set was generated in the same manner, with the exception that the number of the “strong” peaks was selected randomly between 5 and 35.

To mimic experimental diffraction patterns, the peak positions and intensities in the above synthetic patterns were convoluted with a Lorentzian profile function with a half width at half maximum (hwhm) randomly selected between 0.03 and 0.3°. The hwhm of the individual peaks was multiplied by a value selected randomly between 0.95 and 1.0 to increase the variability in the training set.

### Labeled Powder X‐ray Diffraction Patterns for Performance Testing

The 424 labeled PXRD patterns stored by the research group were used to evaluate the prediction performance of the trained classifier. The PXRD patterns were measured using a θ–2θ diffractometer (either MiniFlex 600 or SmartLab (Rigaku)) with Cu Kα radiation. All patterns were analyzed and labeled according to whether an i‐QC phase was present/absent in the sample.

### Powder X‐Ray Diffraction Measurements of Unknown Materials

A total of 161 alloys were synthesized via the arc melting technique during exploratory research on phase diagrams of six different alloy systems, including Al–Si–Ru,^[^ ^36^ ^]^ Al–Fe–Ir, Al–Mn–Ir, Al–Cu–Pt, Al–Pt–Co, and Al–Cu–Ir. The numbers of master ingots were 79, 52, 12, 8, 8, respectively. Diffraction patterns of the as‐cast alloys were measured using a θ–2θ diffractometer SmartLab (Rigaku) with Cu Kα radiation. The alloys were then annealed, and diffraction measurements were performed. The cycle of annealing and diffraction measurement was repeated, resulting in the 440 unlabeled diffraction patterns.

### Scanning Electron Microscopy

The microstructure of the alloy was observed using SEM (SU6600, Hitachi). The composition of each sample was analyzed using an EDX detector (Oxford Instruments) combined with a SEM apparatus. Kikuchi patterns were obtained using the EBSD method and recorded using a 2D detector combined with SEM. The obtained Kikuchi patterns were analyzed using the software EBSD12.^[^ ^54^ ^]^

### Transmission Electron Microscopy

Small pieces of the alloy were lightly crushed in methanol. Fine alloy particles, many of which were thin and sufficiently transparent for carrying out TEM observations, were suspended on a lacey carbon thin film over a copper grid. For this purpose, a drop of the particles suspended in methanol was placed over the carbon film. The grid containing the sample was mounted on a double‐tilt goniometer and inserted into the TEM to obtain the electron diffraction patterns by tilting to various zone axes. A JEOL microscope model 2800 equipped with a field‐emission electron gun operated at 200 kV was used to record the TEM images.

## Conflict of Interest

The authors declare no conflict of interest.

## Supporting information

Supporting Information

## Data Availability

The source codes are available from the GitHub website (https://github.com/SuperspaceLab/ml‐qc‐pxrd). The dataset of the 440 PXRD patterns of unknown materials can be found at the Open Science Framework (https://osf.io/zr2up/). The filenames of the diffraction data labeled with the compositions, alloy systems and the prediction classes are provided in Table S5 in Supplemental Information.
