# Supplementary material for: Deep Learning Enables Rapid Identification of a New Quasicrystal from Multiphase Powder Diffraction Patterns
Source: Adv Sci (Weinh). 2023 Nov 14;11(1):2304546. doi: 10.1002/advs.202304546 (PMC10767418; doi:10.1002/advs.202304546)
Supplement: Supplementary file 1 — Supporting Information [file ADVS-11-2304546-s001.pdf]

ADVANCED SCIENCE
Open Access

Supporting Information

for *Adv. Sci.*, DOI 10.1002/advs.202304546

Deep Learning Enables Rapid Identification of a New Quasicrystal from Multiphase Powder Diffraction Patterns

Hirotaka Uryu, Tsunetomo Yamada, Koichi Kitahara, Alok Singh, Yutaka Iwasaki, Kaoru Kimura, Kanta Hiroki, Naoya Miyao, Asuka Ishikawa, Ryuji Tamura, Satoshi Ohhashi, Chang Liu and Ryo Yoshida*

Figure S1. Ternary diagram for the alloy composition of the Al-Si-Ru samples, of which the PXRD patterns were screened by the binary classifier (model-C). Full circle in bleu, royal-blue, sky-blue, and white colors indicates that the composition of which the diffraction pattern was classified to the prediction class A, B, C, and D, respectively. A color of higher class was selected when the composition of two samples or more are same.

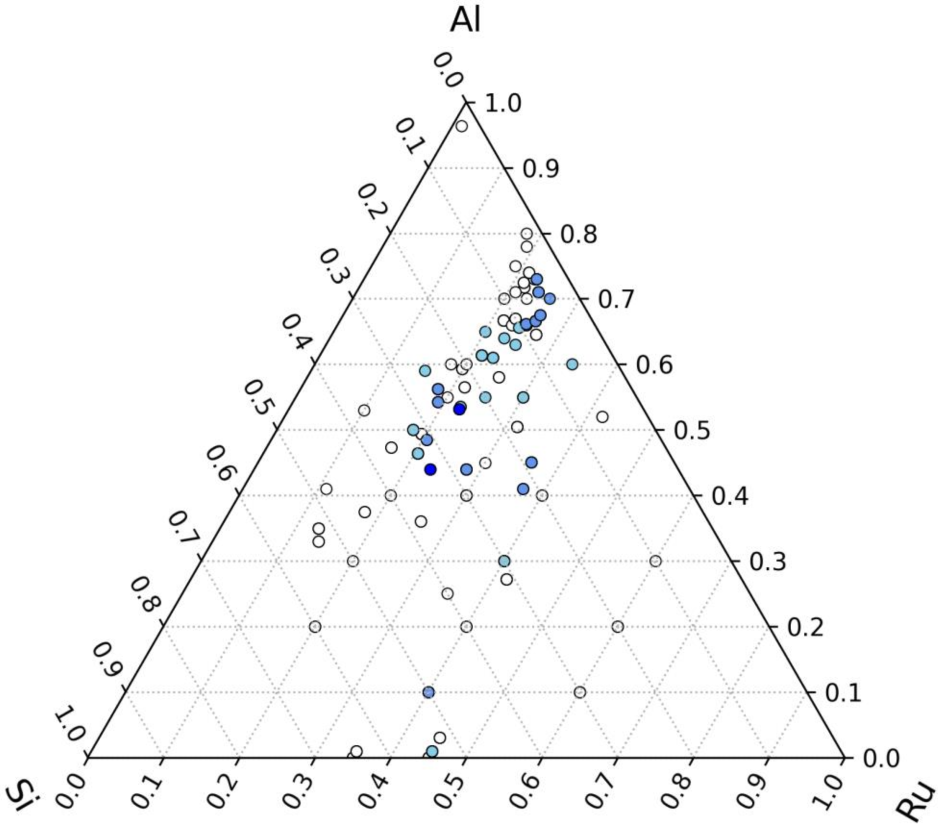

Figure S2. Back-scattered electron scanning image of the ASR(I) sample (a). Electron back-scattered diffraction image taken from the spot indicated by a red triangle (b). The Kikuchi pattern in (b) is indexed as the icosahedral quasicrystal (c). Energy dispersive X-ray analyses of the spots and the analyzed compositions are indicated.

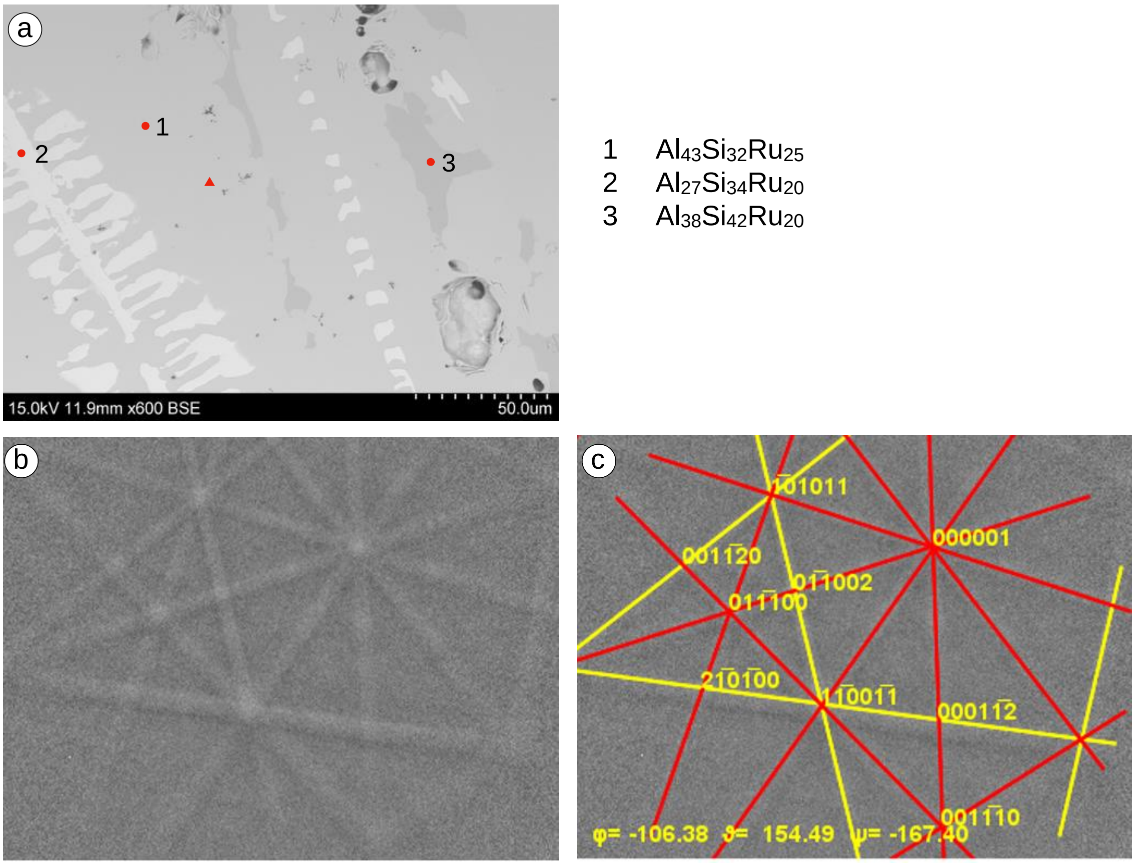

Figure S3. Back-scattered electron scanning image of the ASR(IV) sample (a). Electron back-scattered diffraction image taken from the spot indicated by a red triangle (b). The Kikuchi pattern in (b) is indexed as the icosahedral quasicrystal (c). Energy dispersive X-ray analyses of the spots and the analyzed compositions are indicated.

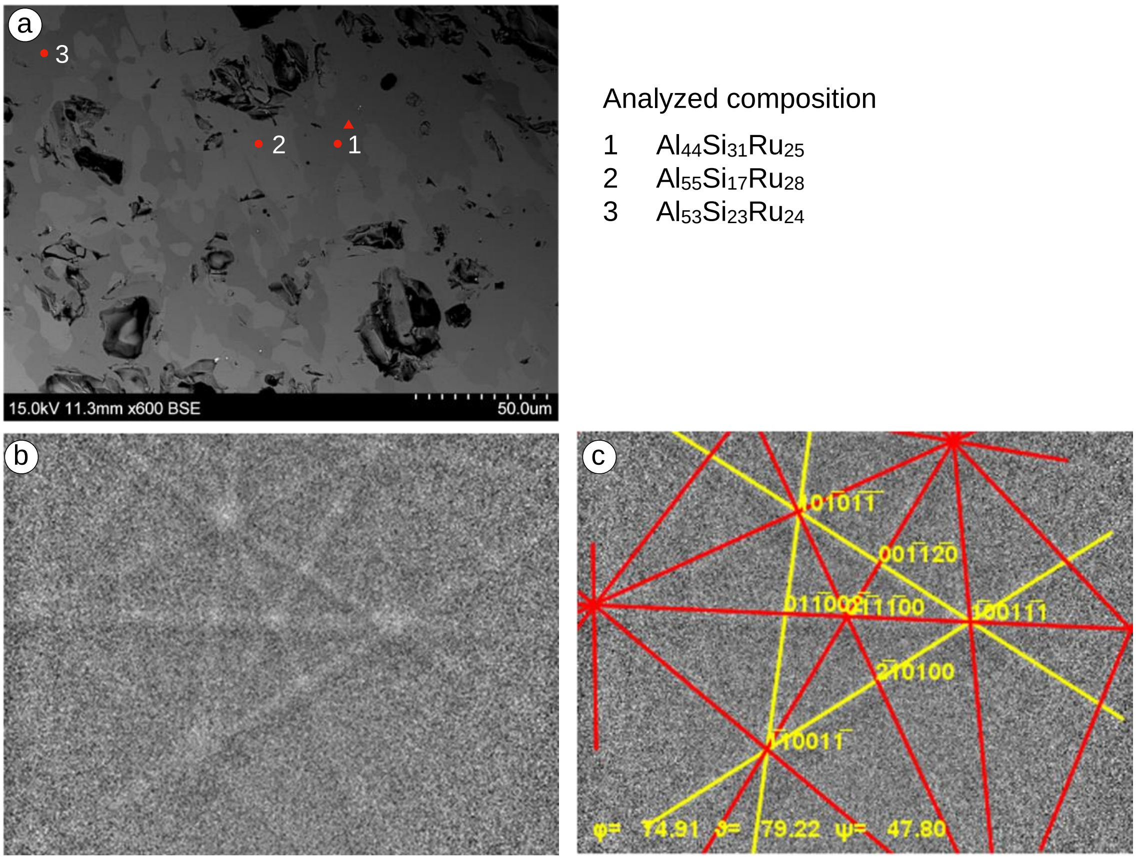

Table S1. Confusion matrix of the trained classifier with respect to (a) 480,000 artificial test datasets and (b) 424 experimental datasets. The normalized confusion matrix is given in parentheses. The accuracy, recall, precision, and F1 values are given at the bottom of each table.

(a) Artificial data

		Prediction	
		**multi-QC**	**others**
**True**	**multi-QC**	237,240 (0.9889)	2,660 (0.011)
	**others**	2,446 (0.010)	237,554 (0.9898)

Recall = 0.9889; **Precision** = 0.9898; **F_1** = 0.9893; **Accuracy** = 0.9893

(b) Experimental data

		Prediction	
		**multi-QC**	**others**
**True**	**multi-QC**	70 (0.958)	3 (0.0411)
	**others**	30 (0.0865)	321 (0.9251)

Recall = 0.9589; **Precision** = 0.7000; **F_1** = 0.8092; **Accuracy** = 0.9222

Table S2. Classification probabilities by the trained classifier for powder X-ray diffraction patterns of known i-QCs of three different families.

i-QC alloys	Structural type	Brave lattice	Probability
Zn-Mg-Sc	Tsai-type	primitive	0.99984
Cd-Mg-Yb	Tsai-type	primitive	0.99994
Ag-In-Yb	Tsai-type	primitive	0.99982
Al-Cu-Fe	Mackay-type	face-centered	0.99893
Al-Cu-Ru	Mackay-type	face-centered	0.99960
Al-Pd-Ru	Mackay-type	face-centered	0.99998
Zn-Mg-Zr	Bergman-type	face-centered	0.99994
Zn-Mg-Hf	Bergman-type	face-centered	0.97267

Table S3. The confusion matrix of the single CNN models trained with (a, b) “single-QC” and (c)

"multi-QC". Prediction performance of the models was evaluated with 10,000 artificial test datasets of (a) "single-QC" and (b, c) "multi-QC". The normalized confusion matrix is given in parentheses. The accuracy, recall, precision values are given at the bottom of each table.

(a) Single CNN model trained with "single-QC" and tested with "single-QC"

		Prediction	
		**multi-QC**	**others**
**True**	**multi-QC**	4,998 (9996)	2 (0.0004)
	**others**	31 (0.0062)	4969 (0.9938)

Recall = 0.9996; **Precision** = 0.9938; **F_1** = 0.9967; **Accuracy** = 0.9967

(b) Single CNN model trained with "single-QC" and tested with "multi-QC"

		Prediction	
		**multi-QC**	**others**
**True**	**multi-QC**	818 (0.1636)	4182 (0.8364)
	**others**	26 (0.0052)	4974 (0.9948)

Recall = 0.1636; **Precision** = 0.9692; **F_1** = 0.2799; **Accuracy** = 0.5792

(c) Single CNN model trained with "multi-QC" and tested with "multi-QC"

		Prediction	
		**multi-QC**	**others**
**True**	**multi-QC**	3777 (0.7554)	1223 (0.2446)
	**others**	847 (0.1694)	4153 (0.8306)

Recall = 0.7554; **Precision** = 0.8168; **F_1** = 0.7849; **Accuracy** = 0.7930

Table S4. List of hyperparameters in the CNN model.

Hyperparameters	Search space

Number of convolutional layers	{1, 2, 3, 4}
Number of kernels in each convolutional layer	{32, 64, …, 96, 128}
Kernel size in each convolutional layer	{10, 15, …, 45, 50}
Stride size in each convolutional layer	{1, 2, 3}
Window size in max pooling	{1, 2, 3}
Stride in max pooling	{1, 2, 3}
Number of fully connected layers	{1, 2, 3}
Number of neurons in each fully connected layer	{500, 1000, …, 2500, 3000}
Dropout rate	{0.0, 0.1, …, 0.4, 0.5}
Optimizer	Adam, SGD

Table S5. Filename of the 440 PXRD pattern labelled with composition, alloy system and the prediction class.

File name	Composition	Alloy system	Prediction class
Al53.2Si24.3Ru22.5_ASR73_0h.txt	Al53.2Si24.3Ru22.5	Al-Si-Ru	A
Al44Si32.8Ru23.2_ASR42_264hpowder.txt	Al44Si32.8Ru23.2	Al-Si-Ru	A
Al44Si32.8Ru23.2_ASR42_1200K_264h_slow.txt	Al44Si32.8Ru23.2	Al-Si-Ru	A
Al44Si32.8Ru23.2_ASR42_1200K_264h.txt	Al44Si32.8Ru23.2	Al-Si-Ru	A
Al44Si28Ru28_ASR68_0h.txt	Al44Si28Ru28	Al-Si-Ru	B
Al80.0Si2.0Ru18_ASR60_93h.txt	Al80.0Si2.0Ru18	Al-Si-Ru	B
Al70Si4Ru26_ASR4_168h.txt	Al70Si4Ru26	Al-Si-Ru	B
Al71Si5Ru24_ASR28_622h.txt	Al71Si5Ru24	Al-Si-Ru	B
Al45.10Si18.88Ru36.02_ASR54_331h.txt	Al45.10Si18.88Ru36.02	Al-Si-Ru	B
Al71Si5Ru24_ASR28_788h.txt	Al71Si5Ru24	Al-Si-Ru	B
Al66.6Si7.6Ru25.8_ASR72_0h.txt	Al66.6Si7.6Ru25.8	Al-Si-Ru	B
Al73.0Si4.2Ru22.8_ASR63_249h.txt	Al73.0Si4.2Ru22.8	Al-Si-Ru	B
Al66.2Si9Ru24.8_ASR48_466h.txt	Al66.2Si9Ru24.8	Al-Si-Ru	B
Al56.28Si25.57Ru18.15_ASR51_580h.txt	Al56.28Si25.57Ru18.15	Al-Si-Ru	B
Al54.3Si26.6Ru19.1_ASR64_0h.txt	Al54.3Si26.6Ru19.1	Al-Si-Ru	B
Al48.5Si31Ru20.5_ASR33_788h.txt	Al48.5Si31Ru20.5	Al-Si-Ru	B
Al10Si50Ru40_ASR15_1205h.txt	Al10Si50Ru40	Al-Si-Ru	B
Al67.5Si6.5Ru26_ASR30_622h.txt	Al67.5Si6.5Ru26	Al-Si-Ru	B

Al41Si22Ru37_ASR36_291h.txt	Al41Si22Ru37	Al-Si-Ru	B
Al10Si50Ru40_ASR15_1200K_459h.txt	Al10Si50Ru40	Al-Si-Ru	B
Al73.0Si4.2Ru22.8_ASR63_249h_rough_polish.txt	Al73.0Si4.2Ru22.8	Al-Si-Ru	C
Al60Si6Ru34_ASR5_168h.txt	Al60Si6Ru34	Al-Si-Ru	C
Al54.3Si26.6Ru19.1_ASR64_249h.txt	Al54.3Si26.6Ru19.1	Al-Si-Ru	C
Al67.5Si6.5Ru26_ASR30_788h.txt	Al67.5Si6.5Ru26	Al-Si-Ru	C
Al45.10Si18.88Ru36.02_ASR54_466h.txt	Al45.10Si18.88Ru36.02	Al-Si-Ru	C
Al63Si12Ru25_ASR8_168h.txt	Al63Si12Ru25	Al-Si-Ru	C
Al50Si32Ru18_ASR35_788h.txt	Al50Si32Ru18	Al-Si-Ru	C
Al48.5Si31Ru20.5_ASR33_622h.txt	Al48.5Si31Ru20.5	Al-Si-Ru	C
Al54.3Si26.6Ru19.1_ASR64_135h.txt	Al54.3Si26.6Ru19.1	Al-Si-Ru	C
Al46.4Si33.2Ru20.4_ASR74_135h.txt	Al46.4Si33.2Ru20.4	Al-Si-Ru	C
Al53.5Si24Ru22.5_ASR32_622h.txt	Al53.5Si24Ru22.5	Al-Si-Ru	C
Al45.10Si18.88Ru36.02_ASR54_899h.txt	Al45.10Si18.88Ru36.02	Al-Si-Ru	C
Al45.10Si18.88Ru36.02_ASR54_580h.txt	Al45.10Si18.88Ru36.02	Al-Si-Ru	C
Al45.10Si18.88Ru36.02_ASR54_740h.txt	Al45.10Si18.88Ru36.02	Al-Si-Ru	C
Al56.28Si25.57Ru18.15_ASR51_899h.txt	Al56.28Si25.57Ru18.15	Al-Si-Ru	C
Al41Si22Ru37_ASR36_788h.txt	Al41Si22Ru37	Al-Si-Ru	C
Al50Si32Ru18_ASR35_291h.txt	Al50Si32Ru18	Al-Si-Ru	C
Al10Si50Ru40_ASR15_1091h.txt	Al10Si50Ru40	Al-Si-Ru	C
Al41Si22Ru37_ASR36_622h.txt	Al41Si22Ru37	Al-Si-Ru	C
Al61Si16Ru23_ASR27_661h.txt	Al61Si16Ru23	Al-Si-Ru	C
Al46.4Si33.2Ru20.4_ASR74_249h.txt	Al46.4Si33.2Ru20.4	Al-Si-Ru	C
Al10Si50Ru40_ASR15_956h.txt	Al10Si50Ru40	Al-Si-Ru	C
Al64Si13Ru23_ASR26_163h.txt	Al64Si13Ru23	Al-Si-Ru	C
Al30Si30Ru40_ASR13_168h.txt	Al30Si30Ru40	Al-Si-Ru	C
Al50Si32Ru18_ASR35_622h.txt	Al50Si32Ru18	Al-Si-Ru	C
Al71Si5Ru24_ASR28_1200K_291h.txt	Al71Si5Ru24	Al-Si-Ru	C
Al56.28Si25.57Ru18.15_ASR51_331h.txt	Al56.28Si25.57Ru18.15	Al-Si-Ru	C
Al53.2Si24.3Ru22.5_ASR73_568h.txt	Al53.2Si24.3Ru22.5	Al-Si-Ru	C
Al59Si26Ru15_ASR58_94h.txt	Al59Si26Ru15	Al-Si-Ru	C
Al30Si30Ru40_ASR13_790h.txt	Al30Si30Ru40	Al-Si-Ru	C

Al65Si15Ru20_ASR17_168h.txt	Al65Si15Ru20	Al-Si-Ru	C
Al61.4Si17.3Ru21.3_ASR71_0h.txt	Al61.4Si17.3Ru21.3	Al-Si-Ru	C
Al61Si16Ru23_ASR27_495h.txt	Al61Si16Ru23	Al-Si-Ru	C
Al71Si5Ru24_ASR28_1200K_291h_slow.txt	Al71Si5Ru24	Al-Si-Ru	C
Al55Si15Ru30_ASR10_499h.txt	Al55Si15Ru30	Al-Si-Ru	C
Al65.65Si10.23Ru24.12_ASR49_466h.txt	Al65.65Si10.23Ru24.12	Al-Si-Ru	C
Al61.4Si17.3Ru21.3_ASR71_409h.txt	Al61.4Si17.3Ru21.3	Al-Si-Ru	C
Al53.2Si24.3Ru22.5_ASR73_730h.txt	Al53.2Si24.3Ru22.5	Al-Si-Ru	C
Al61.4Si17.3Ru21.3_ASR71_568h.txt	Al61.4Si17.3Ru21.3	Al-Si-Ru	C
Al53.2Si24.3Ru22.5_ASR73_409h.txt	Al53.2Si24.3Ru22.5	Al-Si-Ru	C
Al56.28Si25.57Ru18.15_ASR51_466h.txt	Al56.28Si25.57Ru18.15	Al-Si-Ru	C
Al53.2Si24.3Ru22.5_ASR73_135h.txt	Al53.2Si24.3Ru22.5	Al-Si-Ru	C
Al46.4Si33.2Ru20.4_ASR74_568h.txt	Al46.4Si33.2Ru20.4	Al-Si-Ru	C
Al1Si54Ru45_ASR66_730h.txt	Al1Si54Ru45	Al-Si-Ru	C
Al53.5Si24Ru22.5_ASR32_291h.txt	Al53.5Si24Ru22.5	Al-Si-Ru	C
Al61.4Si17.3Ru21.3_ASR71_730h.txt	Al61.4Si17.3Ru21.3	Al-Si-Ru	C
Al61.4Si17.3Ru21.3_ASR71_249h.txt	Al61.4Si17.3Ru21.3	Al-Si-Ru	C
Al10Si50Ru40_ASR15_168h.txt	Al10Si50Ru40	Al-Si-Ru	C
Al46.4Si33.2Ru20.4_ASR74_730h.txt	Al46.4Si33.2Ru20.4	Al-Si-Ru	C
Al1Si54Ru45_ASR66_135h.txt	Al1Si54Ru45	Al-Si-Ru	C
Al66.2Si9Ru24.8_ASR48_331h.txt	Al66.2Si9Ru24.8	Al-Si-Ru	C
Al55Si20Ru25_ASR9_168h.txt	Al55Si20Ru25	Al-Si-Ru	C
Al56.28Si25.57Ru18.15_ASR51_740h.txt	Al56.28Si25.57Ru18.15	Al-Si-Ru	C
Al0Si55Ru45_ASR46_1010h.txt	Al0Si55Ru45	Al-Si-Ru	D
Al0Si55Ru45_ASR46_1170h.txt	Al0Si55Ru45	Al-Si-Ru	D
Al0Si55Ru45_ASR46_1329h.txt	Al0Si55Ru45	Al-Si-Ru	D
Al0Si55Ru45_ASR46_264h.txt	Al0Si55Ru45	Al-Si-Ru	D
Al0Si55Ru45_ASR46_595h.txt	Al0Si55Ru45	Al-Si-Ru	D
Al0Si55Ru45_ASR46_761h.txt	Al0Si55Ru45	Al-Si-Ru	D
Al0Si55Ru45_ASR46_896h.txt	Al0Si55Ru45	Al-Si-Ru	D
Al0Si65Ru35_ASR56_1061h.txt	Al0Si65Ru35	Al-Si-Ru	D
Al0Si65Ru35_ASR56_331h.txt	Al0Si65Ru35	Al-Si-Ru	D

Al0Si65Ru35_ASR56_466h.txt	Al0Si65Ru35	Al-Si-Ru	D
Al0Si65Ru35_ASR56_580h.txt	Al0Si65Ru35	Al-Si-Ru	D
Al0Si65Ru35_ASR56_740h.txt	Al0Si65Ru35	Al-Si-Ru	D
Al0Si65Ru35_ASR56_899h.txt	Al0Si65Ru35	Al-Si-Ru	D
Al10Si30Ru60_ASR39_291h.txt	Al10Si30Ru60	Al-Si-Ru	D
Al10Si30Ru60_ASR39_622h.txt	Al10Si30Ru60	Al-Si-Ru	D
Al10Si30Ru60_ASR39_788h.txt	Al10Si30Ru60	Al-Si-Ru	D
Al10Si30Ru60_ASR39_923h.txt	Al10Si30Ru60	Al-Si-Ru	D
Al10Si50Ru40_ASR15_790h.txt	Al10Si50Ru40	Al-Si-Ru	D
Al1Si54Ru45_ASR66_0h.txt	Al1Si54Ru45	Al-Si-Ru	D
Al1Si54Ru45_ASR66_249h.txt	Al1Si54Ru45	Al-Si-Ru	D
Al1Si54Ru45_ASR66_409h.txt	Al1Si54Ru45	Al-Si-Ru	D
Al1Si54Ru45_ASR66_568h.txt	Al1Si54Ru45	Al-Si-Ru	D
Al1Si64Ru35_ASR67_0h.txt	Al1Si64Ru35	Al-Si-Ru	D
Al1Si64Ru35_ASR67_135h.txt	Al1Si64Ru35	Al-Si-Ru	D
Al1Si64Ru35_ASR67_249h.txt	Al1Si64Ru35	Al-Si-Ru	D
Al1Si64Ru35_ASR67_409h.txt	Al1Si64Ru35	Al-Si-Ru	D
Al1Si64Ru35_ASR67_568h.txt	Al1Si64Ru35	Al-Si-Ru	D
Al1Si64Ru35_ASR67_730h.txt	Al1Si64Ru35	Al-Si-Ru	D
Al20Si20Ru60_ASR38_291h.txt	Al20Si20Ru60	Al-Si-Ru	D
Al20Si20Ru60_ASR38_622h.txt	Al20Si20Ru60	Al-Si-Ru	D
Al20Si20Ru60_ASR38_788h.txt	Al20Si20Ru60	Al-Si-Ru	D
Al20Si20Ru60_ASR38_923h.txt	Al20Si20Ru60	Al-Si-Ru	D
Al20Si40Ru40_ASR14_1200K_459h.txt	Al20Si40Ru40	Al-Si-Ru	D
Al20Si40Ru40_ASR14_168h.txt	Al20Si40Ru40	Al-Si-Ru	D
Al20Si60Ru20_ASR22_163h.txt	Al20Si60Ru20	Al-Si-Ru	D
Al20Si60Ru20_ASR22_495h.txt	Al20Si60Ru20	Al-Si-Ru	D
Al20Si60Ru20_ASR22_661h.txt	Al20Si60Ru20	Al-Si-Ru	D
Al25Si40Ru35_ASR81_0h.txt	Al25Si40Ru35	Al-Si-Ru	D
Al27.22Si31.06Ru41.72_ASR55_1061h.txt	Al27.22Si31.06Ru41.72	Al-Si-Ru	D
Al27.22Si31.06Ru41.72_ASR55_331h.txt	Al27.22Si31.06Ru41.72	Al-Si-Ru	D
Al27.22Si31.06Ru41.72_ASR55_466h.txt	Al27.22Si31.06Ru41.72	Al-Si-Ru	D

Al27.22Si31.06Ru41.72_ASR55_580h.txt	Al27.22Si31.06Ru41.72	Al-Si-Ru	D
Al27.22Si31.06Ru41.72_ASR55_740h.txt	Al27.22Si31.06Ru41.72	Al-Si-Ru	D
Al27.22Si31.06Ru41.72_ASR55_899h.txt	Al27.22Si31.06Ru41.72	Al-Si-Ru	D
Al30Si10Ru60_ASR37_291h.txt	Al30Si10Ru60	Al-Si-Ru	D
Al30Si30Ru40_ASR13_1091h.txt	Al30Si30Ru40	Al-Si-Ru	D
Al30Si30Ru40_ASR13_1200K_459h.txt	Al30Si30Ru40	Al-Si-Ru	D
Al30Si30Ru40_ASR13_956h.txt	Al30Si30Ru40	Al-Si-Ru	D
Al30Si50Ru20_ASR21_163h.txt	Al30Si50Ru20	Al-Si-Ru	D
Al33Si53Ru14_ASR45_264h.txt	Al33Si53Ru14	Al-Si-Ru	D
Al33Si53Ru14_ASR45_595h.txt	Al33Si53Ru14	Al-Si-Ru	D
Al33Si53Ru14_ASR45_761h.txt	Al33Si53Ru14	Al-Si-Ru	D
Al35Si52Ru13_ASR75_0h.txt	Al35Si52Ru13	Al-Si-Ru	D
Al35Si52Ru13_ASR75_135h.txt	Al35Si52Ru13	Al-Si-Ru	D
Al35Si52Ru13_ASR75_249h.txt	Al35Si52Ru13	Al-Si-Ru	D
Al35Si52Ru13_ASR75_409h.txt	Al35Si52Ru13	Al-Si-Ru	D
Al35Si52Ru13_ASR75_568h.txt	Al35Si52Ru13	Al-Si-Ru	D
Al35Si52Ru13_ASR75_730h.txt	Al35Si52Ru13	Al-Si-Ru	D
Al36Si38Ru26_ASR44_264h.txt	Al36Si38Ru26	Al-Si-Ru	D
Al36Si38Ru26_ASR44_595h.txt	Al36Si38Ru26	Al-Si-Ru	D
Al36Si38Ru26_ASR44_761h.txt	Al36Si38Ru26	Al-Si-Ru	D
Al37.5Si44.7Ru17.8_ASR43_1200K_264h.txt	Al37.5Si44.7Ru17.8	Al-Si-Ru	D
Al37.5Si44.7Ru17.8_ASR43_1200K_264h_slow.txt	Al37.5Si44.7Ru17.8	Al-Si-Ru	D
Al37.5Si44.7Ru17.8_ASR43_264hpowder.txt	Al37.5Si44.7Ru17.8	Al-Si-Ru	D
Al3Si52Ru45_ASR47_1056h.txt	Al3Si52Ru45	Al-Si-Ru	D
Al3Si52Ru45_ASR47_1215h.txt	Al3Si52Ru45	Al-Si-Ru	D
Al3Si52Ru45_ASR47_264h.txt	Al3Si52Ru45	Al-Si-Ru	D
Al3Si52Ru45_ASR47_595h.txt	Al3Si52Ru45	Al-Si-Ru	D
Al3Si52Ru45_ASR47_761h.txt	Al3Si52Ru45	Al-Si-Ru	D
Al3Si52Ru45_ASR47_896h.txt	Al3Si52Ru45	Al-Si-Ru	D
Al40Si20Ru40_ASR12_1200K_459h.txt	Al40Si20Ru40	Al-Si-Ru	D
Al40Si20Ru40_ASR12_168h.txt	Al40Si20Ru40	Al-Si-Ru	D
Al40Si30Ru30_ASR7_168h.txt	Al40Si30Ru30	Al-Si-Ru	D

Al40Si30Ru30_ASR7_499h.txt	Al40Si30Ru30	Al-Si-Ru	D
Al40Si30Ru30_ASR7_665h.txt	Al40Si30Ru30	Al-Si-Ru	D
Al40Si40Ru20_ASR20_163h.txt	Al40Si40Ru20	Al-Si-Ru	D
Al41Si48Ru11_ASR59_94h.txt	Al41Si48Ru11	Al-Si-Ru	D
Al44Si28Ru28_ASR68_135h.txt	Al44Si28Ru28	Al-Si-Ru	D
Al44Si28Ru28_ASR68_249h.txt	Al44Si28Ru28	Al-Si-Ru	D
Al44Si28Ru28_ASR68_409h.txt	Al44Si28Ru28	Al-Si-Ru	D
Al44Si28Ru28_ASR68_568h.txt	Al44Si28Ru28	Al-Si-Ru	D
Al44Si28Ru28_ASR68_730h.txt	Al44Si28Ru28	Al-Si-Ru	D
Al45Si25Ru30_ASR6_168h.txt	Al45Si25Ru30	Al-Si-Ru	D
Al46.4Si33.2Ru20.4_ASR74_0h.txt	Al46.4Si33.2Ru20.4	Al-Si-Ru	D
Al46.4Si33.2Ru20.4_ASR74_409h.txt	Al46.4Si33.2Ru20.4	Al-Si-Ru	D
Al47.3Si36.3Ru16.4_ASR65_0h.txt	Al47.3Si36.3Ru16.4	Al-Si-Ru	D
Al47.3Si36.3Ru16.4_ASR65_135h.txt	Al47.3Si36.3Ru16.4	Al-Si-Ru	D
Al47.3Si36.3Ru16.4_ASR65_249h.txt	Al47.3Si36.3Ru16.4	Al-Si-Ru	D
Al48.5Si31Ru20.5_ASR33_291h.txt	Al48.5Si31Ru20.5	Al-Si-Ru	D
Al49.38Si31.27Ru19.35_ASR52_331h.txt	Al49.38Si31.27Ru19.35	Al-Si-Ru	D
Al49.38Si31.27Ru19.35_ASR52_466h.txt	Al49.38Si31.27Ru19.35	Al-Si-Ru	D
Al49.38Si31.27Ru19.35_ASR52_580h.txt	Al49.38Si31.27Ru19.35	Al-Si-Ru	D
Al49.38Si31.27Ru19.35_ASR52_740h.txt	Al49.38Si31.27Ru19.35	Al-Si-Ru	D
Al49.38Si31.27Ru19.35_ASR52_899h.txt	Al49.38Si31.27Ru19.35	Al-Si-Ru	D
Al50.5Si18.0Ru31.5_ASR69_0h.txt	Al50.5Si18.0Ru31.5	Al-Si-Ru	D
Al50.5Si18.0Ru31.5_ASR69_135h.txt	Al50.5Si18.0Ru31.5	Al-Si-Ru	D
Al50.5Si18.0Ru31.5_ASR69_249h.txt	Al50.5Si18.0Ru31.5	Al-Si-Ru	D
Al50.5Si18.0Ru31.5_ASR69_409h.txt	Al50.5Si18.0Ru31.5	Al-Si-Ru	D
Al50.5Si18.0Ru31.5_ASR69_568h.txt	Al50.5Si18.0Ru31.5	Al-Si-Ru	D
Al50.5Si18.0Ru31.5_ASR69_730h.txt	Al50.5Si18.0Ru31.5	Al-Si-Ru	D
Al52Si6Ru42_ASR11_1200K_459h.txt	Al52Si6Ru42	Al-Si-Ru	D
Al52Si6Ru42_ASR11_168h.txt	Al52Si6Ru42	Al-Si-Ru	D
Al53.2Si24.3Ru22.5_ASR73_249h.txt	Al53.2Si24.3Ru22.5	Al-Si-Ru	D
Al53.5Si24Ru22.5_ASR32_788h.txt	Al53.5Si24Ru22.5	Al-Si-Ru	D
Al53Si37Ru10_ASR82_0h.txt	Al53Si37Ru10	Al-Si-Ru	D

Al55Si15Ru30_ASR10_168h.txt	Al55Si15Ru30	Al-Si-Ru	D
Al55Si15Ru30_ASR10_665h.txt	Al55Si15Ru30	Al-Si-Ru	D
Al55Si25Ru20_ASR19_168h.txt	Al55Si25Ru20	Al-Si-Ru	D
Al56.5Si22.0Ru21.5_ASR41_1200K_264h.txt	Al56.5Si22.0Ru21.5	Al-Si-Ru	D
Al56.5Si22.0Ru21.5_ASR41_1200K_264h_slow.txt	Al56.5Si22.0Ru21.5	Al-Si-Ru	D
Al56.5Si22.0Ru21.5_ASR41_264hpowder.txt	Al56.5Si22.0Ru21.5	Al-Si-Ru	D
Al58.0Si16.7Ru25.3_ASR40_1200K_264h.txt	Al58.0Si16.7Ru25.3	Al-Si-Ru	D
Al58.0Si16.7Ru25.3_ASR40_1200K_264h_slow.txt	Al58.0Si16.7Ru25.3	Al-Si-Ru	D
Al58.0Si16.7Ru25.3_ASR40_264hpowder.txt	Al58.0Si16.7Ru25.3	Al-Si-Ru	D
Al59.28Si20.89Ru19.83_ASR53_331h.txt	Al59.28Si20.89Ru19.83	Al-Si-Ru	D
Al59.28Si20.89Ru19.83_ASR53_466h.txt	Al59.28Si20.89Ru19.83	Al-Si-Ru	D
Al59.28Si20.89Ru19.83_ASR53_580h.txt	Al59.28Si20.89Ru19.83	Al-Si-Ru	D
Al59.28Si20.89Ru19.83_ASR53_740h.txt	Al59.28Si20.89Ru19.83	Al-Si-Ru	D
Al59.28Si20.89Ru19.83_ASR53_899h.txt	Al59.28Si20.89Ru19.83	Al-Si-Ru	D
Al60Si20Ru20_ASR18_168h.txt	Al60Si20Ru20	Al-Si-Ru	D
Al60Si22Ru18_ASR34_291h.txt	Al60Si22Ru18	Al-Si-Ru	D
Al61.4Si17.3Ru21.3_ASR71_135h.txt	Al61.4Si17.3Ru21.3	Al-Si-Ru	D
Al61Si16Ru23_ASR27_163h.txt	Al61Si16Ru23	Al-Si-Ru	D
Al64.5Si8.5Ru27_ASR31_1200K_291h.txt	Al64.5Si8.5Ru27	Al-Si-Ru	D
Al64.5Si8.5Ru27_ASR31_1200K_291h_slow_wide.txt	Al64.5Si8.5Ru27	Al-Si-Ru	D
Al64.5Si8.5Ru27_ASR31_622h.txt	Al64.5Si8.5Ru27	Al-Si-Ru	D
Al64.5Si8.5Ru27_ASR31_788h.txt	Al64.5Si8.5Ru27	Al-Si-Ru	D
Al65.65Si10.23Ru24.12_ASR49_331h.txt	Al65.65Si10.23Ru24.12	Al-Si-Ru	D
Al66.6Si7.6Ru25.8_ASR72_135h.txt	Al66.6Si7.6Ru25.8	Al-Si-Ru	D
Al66.6Si7.6Ru25.8_ASR72_249h.txt	Al66.6Si7.6Ru25.8	Al-Si-Ru	D
Al66.6Si7.6Ru25.8_ASR72_409h.txt	Al66.6Si7.6Ru25.8	Al-Si-Ru	D
Al66.6Si7.6Ru25.8_ASR72_568h.txt	Al66.6Si7.6Ru25.8	Al-Si-Ru	D
Al66.6Si7.6Ru25.8_ASR72_730h.txt	Al66.6Si7.6Ru25.8	Al-Si-Ru	D
Al66.7Si11.7Ru21.6_ASR70_0h.txt	Al66.7Si11.7Ru21.6	Al-Si-Ru	D
Al66.7Si11.7Ru21.6_ASR70_135h.txt	Al66.7Si11.7Ru21.6	Al-Si-Ru	D
Al66.7Si11.7Ru21.6_ASR70_249h.txt	Al66.7Si11.7Ru21.6	Al-Si-Ru	D
Al66.7Si11.7Ru21.6_ASR70_409h.txt	Al66.7Si11.7Ru21.6	Al-Si-Ru	D

Al66.7Si11.7Ru21.6_ASR70_568h.txt	Al66.7Si11.7Ru21.6	Al-Si-Ru	D
Al66.7Si11.7Ru21.6_ASR70_730h.txt	Al66.7Si11.7Ru21.6	Al-Si-Ru	D
Al66Si10.5Ru23.5_ASR79_0h.txt	Al66Si10.5Ru23.5	Al-Si-Ru	D
Al66Si11Ru23_ASR80_0h.txt	Al66Si11Ru23	Al-Si-Ru	D
Al66Si9.5Ru24.5_ASR78_0h.txt	Al66Si9.5Ru24.5	Al-Si-Ru	D
Al66Si9Ru25_ASR23_163h.txt	Al66Si9Ru25	Al-Si-Ru	D
Al67.5Si6.5Ru26_ASR30_1200K_291h.txt	Al67.5Si6.5Ru26	Al-Si-Ru	D
Al67Si10Ru23_ASR25_163h.txt	Al67Si10Ru23	Al-Si-Ru	D
Al67Si10Ru23_ASR25_495h.txt	Al67Si10Ru23	Al-Si-Ru	D
Al67Si10Ru23_ASR25_661h.txt	Al67Si10Ru23	Al-Si-Ru	D
Al70Si10Ru20_ASR16_168h.txt	Al70Si10Ru20	Al-Si-Ru	D
Al70Si7Ru23_ASR24_163h.txt	Al70Si7Ru23	Al-Si-Ru	D
Al71.74Si6.50Ru21.76_ASR50_331h.txt	Al71.74Si6.50Ru21.76	Al-Si-Ru	D
Al71.74Si6.50Ru21.76_ASR50_466h.txt	Al71.74Si6.50Ru21.76	Al-Si-Ru	D
Al71Si8Ru21_ASR29_1200K_291h.txt	Al71Si8Ru21	Al-Si-Ru	D
Al72.5Si6.2Ru21.3_ASR61_0h.txt	Al72.5Si6.2Ru21.3	Al-Si-Ru	D
Al72.5Si6.2Ru21.3_ASR61_135h.txt	Al72.5Si6.2Ru21.3	Al-Si-Ru	D
Al72.5Si6.2Ru21.3_ASR61_249h.txt	Al72.5Si6.2Ru21.3	Al-Si-Ru	D
Al73.0Si4.2Ru22.8_ASR63_0h.txt	Al73.0Si4.2Ru22.8	Al-Si-Ru	D
Al73.0Si4.2Ru22.8_ASR63_135h.txt	Al73.0Si4.2Ru22.8	Al-Si-Ru	D
Al73Si4.5Ru22.5_ASR77_0h.txt	Al73Si4.5Ru22.5	Al-Si-Ru	D
Al74.0Si4.7Ru21.3_ASR62_0h.txt	Al74.0Si4.7Ru21.3	Al-Si-Ru	D
Al74.0Si4.7Ru21.3_ASR62_135h.txt	Al74.0Si4.7Ru21.3	Al-Si-Ru	D
Al74.0Si4.7Ru21.3_ASR62_249h.txt	Al74.0Si4.7Ru21.3	Al-Si-Ru	D
Al75Si6Ru19_ASR57_94h.txt	Al75Si6Ru19	Al-Si-Ru	D
Al78Si3Ru19_ASR83_0h.txt	Al78Si3Ru19	Al-Si-Ru	D
Al80.0Si2.0Ru18_ASR60_0h.txt	Al80.0Si2.0Ru18	Al-Si-Ru	D
Al65Pt10Co25_APC6_0h.txt	Al65Pt10Co25	Al-Pt-Co	C
Al60Pt20Co20_APC7_0h.txt	Al60Pt20Co20	Al-Pt-Co	D
Al60Pt30Co10_APC8_0h.txt	Al60Pt30Co10	Al-Pt-Co	D
Al65Pt0Co35_APC3_0h.txt	Al65Pt0Co35	Al-Pt-Co	D
Al70Pt20Co10_APC5_0h.txt	Al70Pt20Co10	Al-Pt-Co	D

Al73Pt0Co27_APC2_0h.txt	Al73Pt0Co27	Al-Pt-Co	D
Al75Pt10Co15_APC4_0h.txt	Al75Pt10Co15	Al-Pt-Co	D
Al79Pt0Co21_APC1_0h.txt	Al79Pt0Co21	Al-Pt-Co	D
Al70Mn30Ir0_AMI12_164h.txt	Al70Mn30Ir0	Al-Mn-Ir	C
Al70Mn30Ir0_AMI12_0h.txt	Al70Mn30Ir0	Al-Mn-Ir	C
Al50Mn50Ir0_AMI9_0h.txt	Al50Mn50Ir0	Al-Mn-Ir	D
Al53.5Mn46.5Ir0_AMI10_0h.txt	Al53.5Mn46.5Ir0	Al-Mn-Ir	D
Al53.5Mn46.5Ir0_AMI10_164h.txt	Al53.5Mn46.5Ir0	Al-Mn-Ir	D
Al53.5Mn46.5Ir0_AMI10_326h.txt	Al53.5Mn46.5Ir0	Al-Mn-Ir	D
Al60Mn10Ir30_AMI8b_0h.txt	Al60Mn10Ir30	Al-Mn-Ir	D
Al60Mn10Ir30_AMI8b_135h.txt	Al60Mn10Ir30	Al-Mn-Ir	D
Al60Mn10Ir30_AMI8b_249h.txt	Al60Mn10Ir30	Al-Mn-Ir	D
Al60Mn10Ir30_AMI8b_409h.txt	Al60Mn10Ir30	Al-Mn-Ir	D
Al60Mn10Ir30_AMI8b_568h.txt	Al60Mn10Ir30	Al-Mn-Ir	D
Al60Mn30Ir10_AMI4b_0h.txt	Al60Mn30Ir10	Al-Mn-Ir	D
Al60Mn30Ir10_AMI4b_135h.txt	Al60Mn30Ir10	Al-Mn-Ir	D
Al60Mn30Ir10_AMI4b_249h.txt	Al60Mn30Ir10	Al-Mn-Ir	D
Al60Mn40Ir0_AMI11_0h.txt	Al60Mn40Ir0	Al-Mn-Ir	D
Al60Mn40Ir0_AMI11_164h.txt	Al60Mn40Ir0	Al-Mn-Ir	D
Al60Mn40Ir0_AMI11_326h.txt	Al60Mn40Ir0	Al-Mn-Ir	D
Al65Mn10Ir25_AMI7b_0h.txt	Al65Mn10Ir25	Al-Mn-Ir	D
Al65Mn10Ir25_AMI7b_135h.txt	Al65Mn10Ir25	Al-Mn-Ir	D
Al65Mn10Ir25_AMI7b_249h.txt	Al65Mn10Ir25	Al-Mn-Ir	D
Al65Mn10Ir25_AMI7b_409h.txt	Al65Mn10Ir25	Al-Mn-Ir	D
Al65Mn10Ir25_AMI7b_568h.txt	Al65Mn10Ir25	Al-Mn-Ir	D
Al65Mn25Ir10_AMI3b_0h.txt	Al65Mn25Ir10	Al-Mn-Ir	D
Al65Mn25Ir10_AMI3b_135h.txt	Al65Mn25Ir10	Al-Mn-Ir	D
Al65Mn25Ir10_AMI3b_249h.txt	Al65Mn25Ir10	Al-Mn-Ir	D
Al70Mn10Ir20_AMI6b_0h.txt	Al70Mn10Ir20	Al-Mn-Ir	D
Al70Mn10Ir20_AMI6b_135h.txt	Al70Mn10Ir20	Al-Mn-Ir	D
Al70Mn10Ir20_AMI6b_249h.txt	Al70Mn10Ir20	Al-Mn-Ir	D
Al70Mn10Ir20_AMI6b_409h.txt	Al70Mn10Ir20	Al-Mn-Ir	D

Al70Mn10Ir20_AMI6b_568h.txt	Al70Mn10Ir20	Al-Mn-Ir	D
Al70Mn20Ir10_AMI2b_0h.txt	Al70Mn20Ir10	Al-Mn-Ir	D
Al70Mn20Ir10_AMI2b_135h.txt	Al70Mn20Ir10	Al-Mn-Ir	D
Al70Mn20Ir10_AMI2b_249h.txt	Al70Mn20Ir10	Al-Mn-Ir	D
Al70Mn20Ir10_AMI2b_409h.txt	Al70Mn20Ir10	Al-Mn-Ir	D
Al70Mn20Ir10_AMI2b_568h.txt	Al70Mn20Ir10	Al-Mn-Ir	D
Al70Mn30Ir0_AMI12_326h.txt	Al70Mn30Ir0	Al-Mn-Ir	D
Al75Mn10Ir15_AMI5b_0h.txt	Al75Mn10Ir15	Al-Mn-Ir	D
Al75Mn10Ir15_AMI5b_135h.txt	Al75Mn10Ir15	Al-Mn-Ir	D
Al75Mn10Ir15_AMI5b_249h.txt	Al75Mn10Ir15	Al-Mn-Ir	D
Al75Mn15Ir10_AMI1b_0h.txt	Al75Mn15Ir10	Al-Mn-Ir	D
Al75Mn15Ir10_AMI1b_135h.txt	Al75Mn15Ir10	Al-Mn-Ir	D
Al75Mn15Ir10_AMI1b_249h.txt	Al75Mn15Ir10	Al-Mn-Ir	D
Al72.7Fe18Ir9.3_AFI47_159h.txt	Al72.7Fe18Ir9.3	Al-Fe-Ir	A
Al72.7Fe18Ir9.3_AFI47_321h.txt	Al72.7Fe18Ir9.3	Al-Fe-Ir	A
Al73.5Fe14.5Ir12_AFI31_757h.txt	Al73.5Fe14.5Ir12	Al-Fe-Ir	B
Al72.5Fe15.5Ir12_AFI13_496h.txt	Al72.5Fe15.5Ir12	Al-Fe-Ir	B
Al72.5Fe15.5Ir12_AFI13_631h.txt	Al72.5Fe15.5Ir12	Al-Fe-Ir	B
Al72Fe16.5Ir11.5_AFI36_483h.txt	Al72Fe16.5Ir11.5	Al-Fe-Ir	B
Al72.5Fe15.5Ir12_AFI13_330h.txt	Al72.5Fe15.5Ir12	Al-Fe-Ir	B
Al75Fe15Ir10_AFI1_163h.txt	Al75Fe15Ir10	Al-Fe-Ir	B
Al72.5Fe21Ir6.5_AFI32_916h.txt	Al72.5Fe21Ir6.5	Al-Fe-Ir	B
Al72.5Fe21Ir6.5_AFI32_757h.txt	Al72.5Fe21Ir6.5	Al-Fe-Ir	B
Al72.5Fe21Ir6.5_AFI32_597h.txt	Al72.5Fe21Ir6.5	Al-Fe-Ir	B
Al73.5Fe14.5Ir12_AFI31_597h.txt	Al73.5Fe14.5Ir12	Al-Fe-Ir	B
Al66Fe22.0Ir12_AFI37_321h.txt	Al66Fe22.0Ir12	Al-Fe-Ir	B
Al63Fe31Ir6_AFI11_631h.txt	Al63Fe31Ir6	Al-Fe-Ir	B
Al73.5Fe14.5Ir12_AFI31_916h.txt	Al73.5Fe14.5Ir12	Al-Fe-Ir	B
Al63Fe31Ir6_AFI11_496h.txt	Al63Fe31Ir6	Al-Fe-Ir	C
Al66Fe22.0Ir12_AFI37_159h.txt	Al66Fe22.0Ir12	Al-Fe-Ir	C
Al74Fe14Ir12_AFI45_321h.txt	Al74Fe14Ir12	Al-Fe-Ir	C
Al73.5Fe14.5Ir12_AFI31_483h.txt	Al73.5Fe14.5Ir12	Al-Fe-Ir	C

Al73Fe12Ir15_AFI18_631h.txt	Al73Fe12Ir15	Al-Fe-Ir	C
Al65Fe25Ir10_AFI3_163h.txt	Al65Fe25Ir10	Al-Fe-Ir	C
Al72.5Fe21Ir6.5_AFI32_483h.txt	Al72.5Fe21Ir6.5	Al-Fe-Ir	C
Al73Fe12Ir15_AFI18_330h.txt	Al73Fe12Ir15	Al-Fe-Ir	C
Al74Fe14Ir12_AFI45_159h.txt	Al74Fe14Ir12	Al-Fe-Ir	C
Al70Fe20Ir10_AFI2_163h.txt	Al70Fe20Ir10	Al-Fe-Ir	C
Al71.3Fe0Ir28.7_AFI43_0h.txt	Al71.3Fe0Ir28.7	Al-Fe-Ir	C
Al70Fe0Ir30_AFI17_496h.txt	Al70Fe0Ir30	Al-Fe-Ir	C
Al75Fe10Ir15_AFI5_163h.txt	Al75Fe10Ir15	Al-Fe-Ir	C
Al73Fe12Ir15_AFI18_496h.txt	Al73Fe12Ir15	Al-Fe-Ir	C
Al70Fe0Ir30_AFI17_631h.txt	Al70Fe0Ir30	Al-Fe-Ir	C
Al50Fe0Ir50_AFI27_483h.txt	Al50Fe0Ir50	Al-Fe-Ir	D
Al56Fe25.5Ir18.5_AFI41_0h.txt	Al56Fe25.5Ir18.5	Al-Fe-Ir	D
Al56Fe25.5Ir18.5_AFI41_159h.txt	Al56Fe25.5Ir18.5	Al-Fe-Ir	D
Al56Fe25.5Ir18.5_AFI41_321h.txt	Al56Fe25.5Ir18.5	Al-Fe-Ir	D
Al60Fe10Ir30_AFI8_163h.txt	Al60Fe10Ir30	Al-Fe-Ir	D
Al60Fe13Ir27_AFI20_330h.txt	Al60Fe13Ir27	Al-Fe-Ir	D
Al60Fe16Ir24_AFI21_330h.txt	Al60Fe16Ir24	Al-Fe-Ir	D
Al60Fe16Ir24_AFI21b_483h.txt	Al60Fe16Ir24	Al-Fe-Ir	D
Al60Fe16Ir24_AFI21b_597h.txt	Al60Fe16Ir24	Al-Fe-Ir	D
Al60Fe16Ir24_AFI21b_757h.txt	Al60Fe16Ir24	Al-Fe-Ir	D
Al60Fe16Ir24_AFI21b_916h.txt	Al60Fe16Ir24	Al-Fe-Ir	D
Al60Fe19Ir21_AFI22_330h.txt	Al60Fe19Ir21	Al-Fe-Ir	D
Al60Fe19Ir21_AFI22b_483h.txt	Al60Fe19Ir21	Al-Fe-Ir	D
Al60Fe19Ir21_AFI22b_597h.txt	Al60Fe19Ir21	Al-Fe-Ir	D
Al60Fe19Ir21_AFI22b_757h.txt	Al60Fe19Ir21	Al-Fe-Ir	D
Al60Fe19Ir21_AFI22b_916h.txt	Al60Fe19Ir21	Al-Fe-Ir	D
Al60Fe22Ir18_AFI23_330h.txt	Al60Fe22Ir18	Al-Fe-Ir	D
Al60Fe25Ir15_AFI24_330h.txt	Al60Fe25Ir15	Al-Fe-Ir	D
Al60Fe25Ir15_AFI24b_483h.txt	Al60Fe25Ir15	Al-Fe-Ir	D
Al60Fe30Ir10_AFI4_163h.txt	Al60Fe30Ir10	Al-Fe-Ir	D
Al60Fe7Ir33_AFI19_330h.txt	Al60Fe7Ir33	Al-Fe-Ir	D

Al60Fe7Ir33_AFI19b_483h.txt	Al60Fe7Ir33	Al-Fe-Ir	D
Al60Fe7Ir33_AFI19b_597h.txt	Al60Fe7Ir33	Al-Fe-Ir	D
Al60Fe7Ir33_AFI19b_757h.txt	Al60Fe7Ir33	Al-Fe-Ir	D
Al60Fe7Ir33_AFI19b_916h.txt	Al60Fe7Ir33	Al-Fe-Ir	D
Al62Fe8.5Ir29.5_AFI46_0h.txt	Al62Fe8.5Ir29.5	Al-Fe-Ir	D
Al62Fe8.5Ir29.5_AFI46_159h.txt	Al62Fe8.5Ir29.5	Al-Fe-Ir	D
Al62Fe8.5Ir29.5_AFI46_321h.txt	Al62Fe8.5Ir29.5	Al-Fe-Ir	D
Al63Fe31Ir6_AFI11_330h.txt	Al63Fe31Ir6	Al-Fe-Ir	D
Al64.5Fe34.0Ir1.5_AFI38_0h.txt	Al64.5Fe34.0Ir1.5	Al-Fe-Ir	D
Al64.5Fe34.0Ir1.5_AFI38_159h.txt	Al64.5Fe34.0Ir1.5	Al-Fe-Ir	D
Al64.5Fe34.0Ir1.5_AFI38_321h.txt	Al64.5Fe34.0Ir1.5	Al-Fe-Ir	D
Al64Fe24Ir12_AFI10_330h.txt	Al64Fe24Ir12	Al-Fe-Ir	D
Al64Fe24Ir12_AFI10_496h.txt	Al64Fe24Ir12	Al-Fe-Ir	D
Al64Fe24Ir12_AFI10_631h.txt	Al64Fe24Ir12	Al-Fe-Ir	D
Al64Fe24Ir12_AFI10_745h.txt	Al64Fe24Ir12	Al-Fe-Ir	D
Al64Fe36Ir0_AFI48_0h.txt	Al64Fe36Ir0	Al-Fe-Ir	D
Al64Fe36Ir0_AFI48_159h.txt	Al64Fe36Ir0	Al-Fe-Ir	D
Al64Fe36Ir0_AFI48_321h.txt	Al64Fe36Ir0	Al-Fe-Ir	D
Al65Fe0Ir35_AFI26_483h.txt	Al65Fe0Ir35	Al-Fe-Ir	D
Al65Fe0Ir35_AFI26_597h.txt	Al65Fe0Ir35	Al-Fe-Ir	D
Al65Fe0Ir35_AFI26_757h.txt	Al65Fe0Ir35	Al-Fe-Ir	D
Al65Fe0Ir35_AFI26_916h.txt	Al65Fe0Ir35	Al-Fe-Ir	D
Al65Fe10Ir25_AFI7_163h.txt	Al65Fe10Ir25	Al-Fe-Ir	D
Al65Fe11.0Ir24_AFI40_0h.txt	Al65Fe11.0Ir24	Al-Fe-Ir	D
Al65Fe11.0Ir24_AFI40_159h.txt	Al65Fe11.0Ir24	Al-Fe-Ir	D
Al65Fe11.0Ir24_AFI40_321h.txt	Al65Fe11.0Ir24	Al-Fe-Ir	D
Al66.67Fe33.33Ir0_AFI33_483h.txt	Al66.67Fe33.33Ir0	Al-Fe-Ir	D
Al66Fe22.0Ir12_AFI37_0h.txt	Al66Fe22.0Ir12	Al-Fe-Ir	D
Al69Fe31Ir0_AFI9_1064h.txt	Al69Fe31Ir0	Al-Fe-Ir	D
Al69Fe31Ir0_AFI9_330h.txt	Al69Fe31Ir0	Al-Fe-Ir	D
Al69Fe31Ir0_AFI9_496h.txt	Al69Fe31Ir0	Al-Fe-Ir	D
Al69Fe31Ir0_AFI9_631h.txt	Al69Fe31Ir0	Al-Fe-Ir	D

Al69Fe31Ir0_AFI9_745h.txt	Al69Fe31Ir0	Al-Fe-Ir	D
Al69Fe31Ir0_AFI9_905h.txt	Al69Fe31Ir0	Al-Fe-Ir	D
Al70Fe0Ir30_AFI17_330h.txt	Al70Fe0Ir30	Al-Fe-Ir	D
Al70Fe1.2Ir28.8_AFI42_0h.txt	Al70Fe1.2Ir28.8	Al-Fe-Ir	D
Al70Fe1.2Ir28.8_AFI42_159h.txt	Al70Fe1.2Ir28.8	Al-Fe-Ir	D
Al70Fe1.2Ir28.8_AFI42_321h.txt	Al70Fe1.2Ir28.8	Al-Fe-Ir	D
Al70Fe10Ir20_AFI6_163h.txt	Al70Fe10Ir20	Al-Fe-Ir	D
Al71.3Fe0Ir28.7_AFI43_159h.txt	Al71.3Fe0Ir28.7	Al-Fe-Ir	D
Al71.3Fe0Ir28.7_AFI43_321h.txt	Al71.3Fe0Ir28.7	Al-Fe-Ir	D
Al71.43Fe28.57Ir0_AFI28_483h.txt	Al71.43Fe28.57Ir0	Al-Fe-Ir	D
Al71.43Fe28.57Ir0_AFI34_483h.txt	Al71.43Fe28.57Ir0	Al-Fe-Ir	D
Al71Fe22Ir7_AFI15_330h.txt	Al71Fe22Ir7	Al-Fe-Ir	D
Al71Fe22Ir7_AFI15_496h.txt	Al71Fe22Ir7	Al-Fe-Ir	D
Al71Fe22Ir7_AFI15_631h.txt	Al71Fe22Ir7	Al-Fe-Ir	D
Al72.7Fe18Ir9.3_AFI47_0h.txt	Al72.7Fe18Ir9.3	Al-Fe-Ir	D
Al72Fe0Ir28_AFI25_483h.txt	Al72Fe0Ir28	Al-Fe-Ir	D
Al74Fe14Ir12_AFI45_0h.txt	Al74Fe14Ir12	Al-Fe-Ir	D
Al74Fe26Ir0_AFI35_483h.txt	Al74Fe26Ir0	Al-Fe-Ir	D
Al74Fe26Ir0_AFI35_597h.txt	Al74Fe26Ir0	Al-Fe-Ir	D
Al74Fe26Ir0_AFI35_757h.txt	Al74Fe26Ir0	Al-Fe-Ir	D
Al74Fe26Ir0_AFI35_916h.txt	Al74Fe26Ir0	Al-Fe-Ir	D
Al75.6Fe9.6Ir14.8_AFI39_0h.txt	Al75.6Fe9.6Ir14.8	Al-Fe-Ir	D
Al75.6Fe9.6Ir14.8_AFI39_159h.txt	Al75.6Fe9.6Ir14.8	Al-Fe-Ir	D
Al75.6Fe9.6Ir14.8_AFI39_321h.txt	Al75.6Fe9.6Ir14.8	Al-Fe-Ir	D
Al75Fe12Ir13_AFI16_330h.txt	Al75Fe12Ir13	Al-Fe-Ir	D
Al75Fe12Ir13_AFI16_496h.txt	Al75Fe12Ir13	Al-Fe-Ir	D
Al75Fe12Ir13_AFI16_631h.txt	Al75Fe12Ir13	Al-Fe-Ir	D
Al75Fe18Ir7_AFI14_330h.txt	Al75Fe18Ir7	Al-Fe-Ir	D
Al75Fe18Ir7_AFI14_496h.txt	Al75Fe18Ir7	Al-Fe-Ir	D
Al75Fe18Ir7_AFI14_631h.txt	Al75Fe18Ir7	Al-Fe-Ir	D
Al75Fe9Ir16_AFI12_1064h.txt	Al75Fe9Ir16	Al-Fe-Ir	D
Al75Fe9Ir16_AFI12_330h.txt	Al75Fe9Ir16	Al-Fe-Ir	D

Al75Fe9Ir16_AFI12_496h.txt	Al75Fe9Ir16	Al-Fe-Ir	D
Al75Fe9Ir16_AFI12_631h.txt	Al75Fe9Ir16	Al-Fe-Ir	D
Al75Fe9Ir16_AFI12_745h.txt	Al75Fe9Ir16	Al-Fe-Ir	D
Al75Fe9Ir16_AFI12_905h.txt	Al75Fe9Ir16	Al-Fe-Ir	D
Al76.8Fe4.7Ir18.5_AFI44_0h.txt	Al76.8Fe4.7Ir18.5	Al-Fe-Ir	D
Al76.8Fe4.7Ir18.5_AFI44_159h.txt	Al76.8Fe4.7Ir18.5	Al-Fe-Ir	D
Al76.8Fe4.7Ir18.5_AFI44_321h.txt	Al76.8Fe4.7Ir18.5	Al-Fe-Ir	D
Al77Fe10Ir13_AFI30_483h.txt	Al77Fe10Ir13	Al-Fe-Ir	D
Al77Fe10Ir13_AFI30_597h.txt	Al77Fe10Ir13	Al-Fe-Ir	D
Al77Fe10Ir13_AFI30_757h.txt	Al77Fe10Ir13	Al-Fe-Ir	D
Al77Fe10Ir13_AFI30_916h.txt	Al77Fe10Ir13	Al-Fe-Ir	D
Al77Fe7Ir16_AFI29_483h.txt	Al77Fe7Ir16	Al-Fe-Ir	D
Al77Fe7Ir16_AFI29_597h.txt	Al77Fe7Ir16	Al-Fe-Ir	D
Al77Fe7Ir16_AFI29_757h.txt	Al77Fe7Ir16	Al-Fe-Ir	D
Al77Fe7Ir16_AFI29_916h.txt	Al77Fe7Ir16	Al-Fe-Ir	D
Al50Cu40Pt10_ACP7_0h.txt	Al50Cu40Pt10	Al-Cu-Pt	D
Al50Cu40Pt10_ACP7_164h.txt	Al50Cu40Pt10	Al-Cu-Pt	D
Al55Cu0Pt45_ACP3_0h.txt	Al55Cu0Pt45	Al-Cu-Pt	D
Al55Cu0Pt45_ACP3_164h.txt	Al55Cu0Pt45	Al-Cu-Pt	D
Al55Cu0Pt45_ACP3_326h.txt	Al55Cu0Pt45	Al-Cu-Pt	D
Al55Cu15Pt30_ACP8_0h.txt	Al55Cu15Pt30	Al-Cu-Pt	D
Al55Cu15Pt30_ACP8_164h.txt	Al55Cu15Pt30	Al-Cu-Pt	D
Al55Cu15Pt30_ACP8_326h.txt	Al55Cu15Pt30	Al-Cu-Pt	D
Al55Cu30Pt15_ACP6_0h.txt	Al55Cu30Pt15	Al-Cu-Pt	D
Al55Cu30Pt15_ACP6_164h.txt	Al55Cu30Pt15	Al-Cu-Pt	D
Al60Cu20Pt20_ACP5_0h.txt	Al60Cu20Pt20	Al-Cu-Pt	D
Al60Cu20Pt20_ACP5_164h.txt	Al60Cu20Pt20	Al-Cu-Pt	D
Al63Cu0Pt37_ACP2_0h.txt	Al63Cu0Pt37	Al-Cu-Pt	D
Al63Cu0Pt37_ACP2_164h.txt	Al63Cu0Pt37	Al-Cu-Pt	D
Al63Cu0Pt37_ACP2_326h.txt	Al63Cu0Pt37	Al-Cu-Pt	D
Al65Cu10Pt25_ACP4_0h.txt	Al65Cu10Pt25	Al-Cu-Pt	D
Al65Cu10Pt25_ACP4_164h.txt	Al65Cu10Pt25	Al-Cu-Pt	D

Al65Cu10Pt25_ACP4_326h.txt	Al65Cu10Pt25	Al-Cu-Pt	D
Al70Cu0Pt30_ACP1_0h.txt	Al70Cu0Pt30	Al-Cu-Pt	D
Al70Cu0Pt30_ACP1_164h.txt	Al70Cu0Pt30	Al-Cu-Pt	D
Al70Cu0Pt30_ACP1_326h.txt	Al70Cu0Pt30	Al-Cu-Pt	D
Al65Cu12Ir23_No03.txt	Al65Cu12Ir23	Al-Cu-Ir	D
Al66Cu11Ir23_No18.txt	Al66Cu11Ir23	Al-Cu-Ir	D
